# Supplementary material for: Analysis of vibronic coupling in a 4f molecular magnet with FIRMS
Source: Nat Commun. 2022 Feb 11;13:825. doi: 10.1038/s41467-022-28352-2 (PMC8837795; doi:10.1038/s41467-022-28352-2)
Supplement: Supplementary file 1 — Supplementary Information [file 41467_2022_28352_MOESM1_ESM.pdf]

Supplementary Information for: Analysis of vibronic coupling in a 4f molecular magnet with FIRMS

Jon G. C. Kragskow[a,†], Jonathan Marbey[b,c,†], Christian D. Buch,[d] Joscha Nehrkorn[b], Mykhaylo Ozerov[b], Stergios Piligkos[d,*], Stephen Hill[b,c,*] and Nicholas F. Chilton[a,*]

[a] Department of Chemistry, School of Natural Sciences, University of Manchester, Oxford Road, Manchester, M13 9PL, United Kingdom

[b] National High Magnetic Field Laboratory, Tallahassee, Florida, 32310, United States of America

[c] Department of Physics, Florida State University, Tallahassee, Florida, 32306, United States of America

[d] Department of Chemistry, University of Copenhagen, DK-2100 Copenhagen, Denmark

[†] These two authors contributed equally to this work.

Email: piligkos@chem.ku.dk; shill@magnet.fsu.edu; nicholas.chilton@manchester.ac.uk

Contents

Experimental electronic structure 2
Ab initio electronic structure and vibrational modes 3
"Toy model" Hamiltonian 14
Ab initio vibronic coupling and simulation of FIRMS maps 18
Experimental and theoretical FIRMS maps and analysis 26
References 44

Experimental electronic structure

Supplementary Table 1: CFPs obtained from fits to the experimental emission spectrum of **1**.[1]

CFP	Experiment (cm^{-1})
B_2^0	-11.13(7)
B_4^{-3}	0
B_4^0	0.153(3)
B_4^3	8.92(4)
B_6^{-6}	0.101(7)
B_6^{-3}	0.07(3)
B_0^6	0.0071(2)
B_3^6	-0.060(3)
B_6^6	0.034(2)

Supplementary Table 2: Doublets of J = 7/2 multiplet of **1**: CF energies from experiment, from parameterised model of experiment (main text Equation 1), and principal g-values and compositions of CF wave functions of model in terms of pure $|J, m_J\rangle$ states.

Observed energy (cm^{-1})	Model CF energies (cm^{-1})	$g_\perp$	$g_{\|\|}$	CF wave function composition	$\langle \hat{J}_z \rangle$
0	0	2.95	4.33	58.333% $\|\pm 7/2\rangle$ + 29.778% $\|\pm 1/2\rangle$ + 11.889% $\|\mp 5/2\rangle$	±1.895
474	464.2	2.39	0.30	52.305% $\|\pm 5/2\rangle$ + 31.374% $\|\mp 7/2\rangle$ + 16.321% $\|\mp 1/2\rangle$	±0.130
745	736.6	0.00	3.43	100% $\|\pm 3/2\rangle$	±1.500
920	912.7	3.62	0.61	53.901% $\|\mp 1/2\rangle$ + 35.806% $\|\pm 5/2\rangle$ + 10.293% $\|\mp 7/2\rangle$	±0.256

Ab initio electronic structure and vibrational modes

Supplementary Table 3: CASSCF-CASPT2-SO results for the $^2F_{7/2}$ ground term of **1** calculated using the x-ray crystallographic structure.

**Energy (cm^{-1})**	$g_\perp$	$g_{\|\|}$	**CF wave function composition**	$\langle \hat{J}_z \rangle$
0.0	2.69	4.98	68.736 % $\|\pm 7/2\rangle$ + 18.706 % $\|\pm 1/2\rangle$ + 12.558 % $\|\mp 5/2\rangle$	±2.185
436.7	2.25	3.38	83.018 % $\|\pm 5/2\rangle$ + 16.823 % $\|\mp 7/2\rangle$ + 0.159 % $\|\mp 1/2\rangle$	±1.486
729.9	0.01	3.39	100 % $\|\pm 3/2\rangle$	±1.500
905.3	4.18	1.83	81.134 % $\|\pm 1/2\rangle$ + 14.441 % $\|\pm 7/2\rangle$ + 4.424 % $\|\mp 5/2\rangle$	±0.801

Supplementary Table 4: CASSCF-SO results for the $^2F_{7/2}$ ground term of **1** calculated using the x-ray crystallographic structure.

**Energy (cm^{-1})**	$g_\perp$	$g_{\|\|}$	**CF wave function composition**	$\langle \hat{J}_z \rangle$
0.0	2.51	5.32	71.960 % $\|\pm 7/2\rangle$ + 17.149 % $\|\pm 1/2\rangle$ + 10.891 % $\|\mp 5/2\rangle$	±2.332
293.9	2.15	3.55	84.127 % $\|\pm 5/2\rangle$ + 15.356 % $\|\mp 7/2\rangle$ + 0.517 % $\|\mp 1/2\rangle$	±1.563
541.7	0.00	3.39	100 % $\|\pm 3/2\rangle$	±1.500
636.6	4.23	1.68	82.334 % $\|\pm 1/2\rangle$ + 12.684 % $\|\pm 7/2\rangle$ + 4.982 % $\|\mp 5/2\rangle$	±0.731

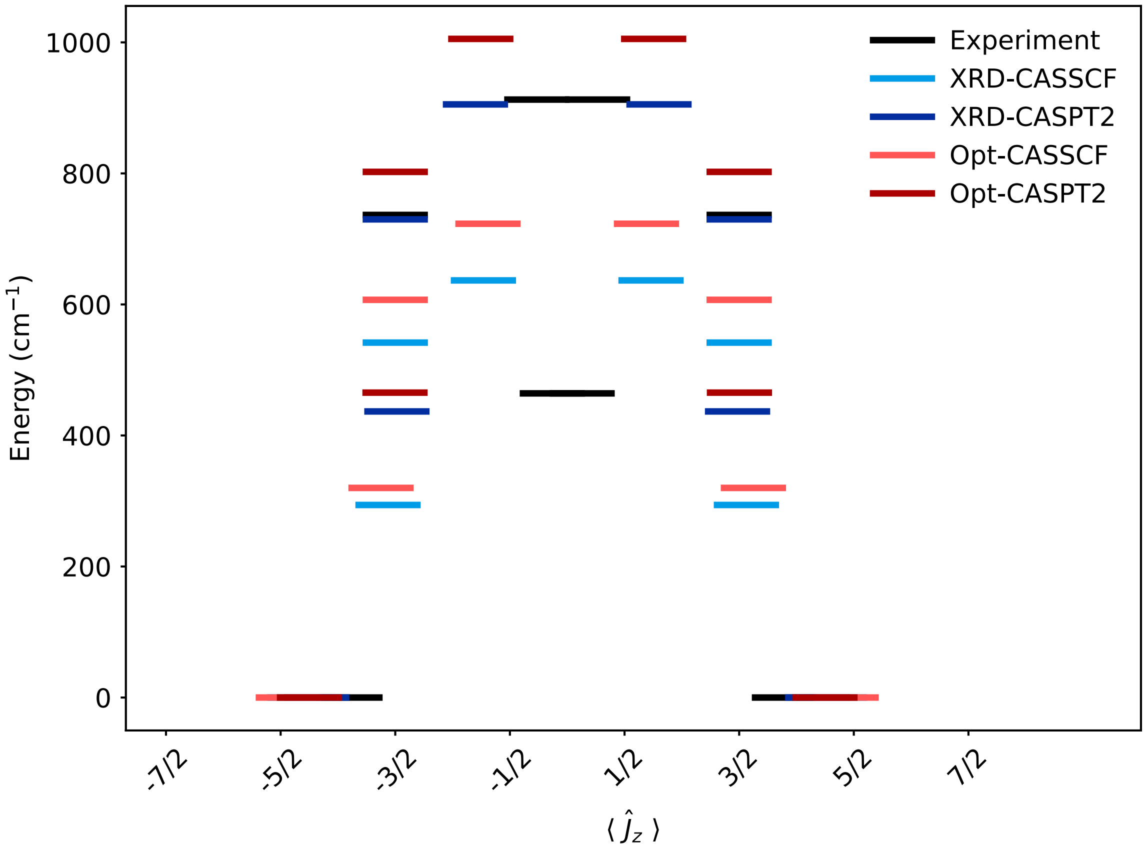

Supplementary Figure 1: Energies and $\langle \hat{J}_z \rangle$ expectation values of the $^2F_{7/2}$ states of **1** obtained from the experimental CFPs in Supplementary Table 2 (black), the CASSCF-SO CFPs X-Ray (XRD) crystallographic structure in Supplementary Table 4 (light blue), and the CASSCF-CASPT2-SO CFPs of **1** in Supplementary Table 3 (dark blue), the CASSCF-SO CFPs of the optimised ($\mathbf{1}_{opt}$) structure in Supplementary Table 7 (light red), and the CASSCF-CASPT2-SO CFPs of $\mathbf{1}_{opt}$ in Supplementary Table 8 (dark red).

Supplementary Table 5: Coordinates of $\mathbf{1}_{opt}$ calculated with DFT using the PBE0 functional, see Methods.

**Atom**	**x (Å)**	**y (Å)**	**z (Å)**
Yb	0.000000	0.000000	0.167551
N	0.000000	0.000000	2.969546
N	-0.679435	2.087931	1.147990
O	-1.738881	0.577725	-0.934359
O	0.369116	-1.794778	-0.934359
O	1.369765	1.217053	-0.934359
C	0.000000	2.392490	2.390990

H	1.091101	2.352570	2.223290
H	-0.243583	3.402480	2.768283
C	-2.445422	1.618309	-1.218309
C	-3.362648	1.594206	-2.301797
H	-3.428500	0.668061	-2.875191
C	-4.127953	2.697898	-2.620804
H	-4.817380	2.642871	-3.467543
C	-4.038677	3.891285	-1.878245
H	-4.649999	4.755585	-2.140554
C	-3.164966	3.941601	-0.812501
H	-3.080837	4.855275	-0.215902
C	-2.359906	2.834241	-0.462058
C	-1.508764	2.963054	0.684773
H	-1.603744	3.923607	1.224104
C	-0.407810	1.342300	3.413145
H	0.001879	1.571607	4.415725
H	-1.504741	1.364693	3.497102
C	-2.071957	-1.196245	2.390990
H	-2.582936	-0.231364	2.223290
H	-2.824842	-1.912189	2.768283
N	-1.468484	-1.632373	1.147990
C	-1.350613	-5.443239	-1.878245
H	-1.793458	-6.404809	-2.140554
C	-1.831044	-4.711741	-0.812501
H	-2.664373	-5.095720	-0.215902
C	-1.274572	-3.460858	-0.462058
C	-1.811698	-2.788155	0.684773
H	-2.596071	-3.350686	1.224104
C	-0.958561	-1.024323	3.413145
H	-1.361991	-0.784176	4.415725
H	-0.429489	-1.985491	3.497102
C	-0.178785	-2.926952	-1.218309
C	0.300701	-3.709241	-2.301797
H	1.135692	-3.303199	-2.875191
C	-0.272472	-4.923861	-2.620804

H	0.119896	-5.493409	-3.467543
C	2.071957	-1.196245	2.390990
H	1.491835	-2.121206	2.223290
H	3.068425	-1.490291	2.768283
N	2.147919	-0.455558	1.147990
C	5.389290	1.551954	-1.878245
H	6.443457	1.649225	-2.140554
C	4.996010	0.770140	-0.812501
H	5.745210	0.240445	-0.215902
C	3.634477	0.626618	-0.462058
C	3.320462	-0.174899	0.684773
H	4.199815	-0.572920	1.224104
C	1.366371	-0.317976	3.413145
H	1.360112	-0.787430	4.415725
H	1.934230	0.620797	3.497102
C	2.624208	1.308643	-1.218309
C	3.061947	2.115036	-2.301797
H	2.292808	2.635137	-2.875191
C	4.400425	2.225963	-2.620804

Supplementary Table 6: Vibrational mode numbers (*j*), irreducible representations (Mulliken Symbols), energies, transition intensities, and vibronic coupling strength (S_j) of the vibrational modes of **1**$_{\mathbf{opt}}$. Vibrational modes observed in the main region of the FIRMS map, and discussed in the main text, are highlighted. Note that C_3 is an Abelian group, hence the E representation is not strictly irreducible, but we still refer to it as such.

Mode Number (*j*)	IRREP	Energy (cm^{-1})	Transition Intensity (km mol^{-1})	S_j (cm^{-1})	Mode Number (*j*)	IRREP	Energy (cm^{-1})	Transition Intensity (km mol^{-1})	S_j (cm^{-1})
1	E	17.049	0.270	0.232	91	A	1058.866	2.934	0.010
2	E	17.049	0.270	0.232	92	E	1070.501	22.695	0.118
3	A	21.963	0.022	0.399	93	E	1070.501	22.696	0.118
4	E	48.448	0.401	0.384	94	A	1092.938	1.535	0.079
5	E	48.449	0.401	0.384	95	E	1112.483	23.902	0.208
6	A	61.248	0.002	0.370	96	E	1112.483	23.901	0.208
7	E	98.981	1.759	0.827	97	A	1118.476	18.408	0.207
8	E	98.981	1.759	0.827	98	E	1150.153	16.466	0.105
9	A	102.101	5.546	0.435	99	E	1150.153	16.466	0.105
10	A	105.188	2.419	0.498	100	A	1151.537	3.958	0.088

11	E	121.533	1.798	0.521	101	E	1164.854	7.382	0.219
12	E	121.534	1.798	0.521	102	E	1164.854	7.382	0.220
13	A	143.349	0.277	0.521	103	A	1165.943	37.694	0.001
14	E	168.810	2.013	0.784	104	E	1173.738	20.455	0.038
15	E	168.811	2.013	0.783	105	E	1173.738	20.454	0.039
16	A	194.872	1.063	0.259	106	A	1223.420	1.307	0.047
17	E	201.390	2.894	0.503	107	E	1242.242	28.568	0.178
18	E	201.390	2.893	0.504	108	E	1242.242	28.569	0.179
19	A	206.609	5.651	0.251	109	A	1246.953	78.896	0.061
20	E	207.233	26.085	0.549	110	E	1259.564	12.173	0.222
21	E	207.234	26.086	0.549	111	E	1259.564	12.175	0.223
22	E	243.421	10.605	0.705	112	A	1265.640	47.647	0.075
23	E	243.421	10.605	0.704	113	E	1266.950	15.140	0.140
24	A	243.713	4.543	0.398	114	E	1266.950	15.139	0.139
25	A	302.226	4.527	0.370	115	A	1301.195	22.197	0.059
26	E	304.782	19.765	0.935	116	E	1304.526	15.162	0.062
27	E	304.782	19.765	0.935	117	E	1304.526	15.164	0.062
28	A	339.877	0.054	0.263	118	E	1348.067	14.481	0.165
29	E	354.097	2.821	0.196	119	E	1348.067	14.482	0.164
30	E	354.097	2.821	0.197	120	A	1356.417	50.598	0.153
31	A	363.366	3.945	0.457	121	E	1390.003	77.962	0.527
32	E	376.891	26.744	0.401	122	E	1390.003	77.962	0.527
33	E	376.891	26.745	0.402	123	A	1393.668	0.311	0.038
34	A	405.921	13.149	0.576	124	E	1397.156	39.141	0.265
35	E	413.150	26.322	0.989	125	E	1397.156	39.143	0.265
36	E	413.150	26.321	0.989	126	A	1398.842	0.516	0.067
37	A	444.750	0.285	0.291	127	A	1407.342	0.095	0.032
38	E	445.645	2.370	0.415	128	E	1407.378	45.776	0.101
39	E	445.645	2.371	0.415	129	E	1407.378	45.775	0.101
40	A	472.671	16.744	0.065	130	E	1428.371	31.732	0.053
41	E	477.090	0.993	0.121	131	E	1428.371	31.732	0.054
42	E	477.091	0.992	0.121	132	A	1429.216	13.744	0.010
43	A	516.190	7.281	0.064	133	E	1456.936	10.928	0.100
44	E	520.657	1.425	0.549	134	E	1456.936	10.927	0.100
45	E	520.657	1.425	0.549	135	A	1457.860	41.731	0.082
46	A	563.246	0.060	0.103	136	E	1466.478	10.459	0.114
47	E	564.025	0.152	0.236	137	E	1466.478	10.460	0.114
48	E	564.025	0.152	0.235	138	A	1471.148	6.904	0.082
49	E	582.008	9.752	0.460	139	E	1506.969	16.695	0.188
50	E	582.008	9.752	0.460	140	E	1506.969	16.695	0.187
51	A	586.006	5.708	0.175	141	A	1507.485	14.102	0.052
52	E	603.156	39.954	0.359	142	E	1526.602	433.168	1.147
53	E	603.156	39.953	0.359	143	E	1526.602	433.180	1.147

54	A	605.684	1.054	0.033	144	A	1536.935	161.499	0.049
55	E	652.681	10.203	0.283	145	E	1602.423	110.761	0.289
56	E	652.681	10.203	0.283	146	E	1602.423	110.758	0.289
57	A	653.219	14.113	0.142	147	A	1603.415	240.739	0.022
58	E	762.003	30.225	0.022	148	E	1679.672	5.520	0.434
59	E	762.003	30.226	0.022	149	E	1679.672	5.521	0.434
60	A	762.023	36.891	0.044	150	A	1680.433	6.621	0.007
61	A	767.926	7.513	0.352	151	E	1701.208	933.492	0.280
62	A	785.476	15.297	0.075	152	E	1701.208	933.489	0.281
63	E	785.570	14.472	0.107	153	A	1716.376	597.625	0.025
64	E	785.570	14.472	0.107	154	E	3011.772	3.033	0.030
65	E	821.184	23.449	0.363	155	E	3011.772	3.032	0.030
66	E	821.184	23.450	0.363	156	A	3014.678	23.269	0.075
67	A	831.122	0.027	0.067	157	E	3025.158	45.235	0.076
68	E	884.926	16.338	0.067	158	E	3025.158	45.231	0.077
69	E	884.926	16.338	0.067	159	A	3028.039	188.955	0.045
70	A	885.703	9.753	0.053	160	E	3047.653	51.683	0.111
71	E	888.620	4.982	0.253	161	E	3047.653	51.683	0.111
72	E	888.620	4.981	0.252	162	A	3048.113	42.433	0.031
73	E	912.529	46.795	0.256	163	E	3076.895	28.874	0.057
74	E	912.529	46.797	0.256	164	E	3076.895	28.871	0.056
75	A	916.245	3.304	0.038	165	A	3077.141	13.919	0.020
76	A	931.696	25.770	0.087	166	A	3110.499	1.940	0.022
77	E	953.927	0.723	0.033	167	E	3116.819	32.695	0.029
78	E	953.928	0.723	0.033	168	E	3116.819	32.699	0.029
79	A	954.069	0.470	0.015	169	E	3179.543	13.829	0.018
80	E	961.663	0.187	0.112	170	E	3179.543	13.828	0.017
81	E	961.663	0.187	0.112	171	A	3179.593	5.148	0.004
82	E	1001.401	0.256	0.007	172	E	3195.542	6.700	0.014
83	E	1001.401	0.256	0.007	173	E	3195.542	6.701	0.014
84	A	1001.447	0.204	0.004	174	A	3195.574	19.944	0.001
85	A	1010.930	5.017	0.012	175	E	3225.252	17.020	0.005
86	E	1012.553	1.503	0.088	176	E	3225.252	17.019	0.006
87	E	1012.553	1.503	0.089	177	A	3225.287	2.224	0.003
88	A	1041.994	13.635	0.038	178	E	3231.521	15.861	0.013
89	E	1057.492	16.170	0.048	179	E	3231.521	15.862	0.014
90	E	1057.492	16.171	0.047	180	A	3231.581	12.413	0.007

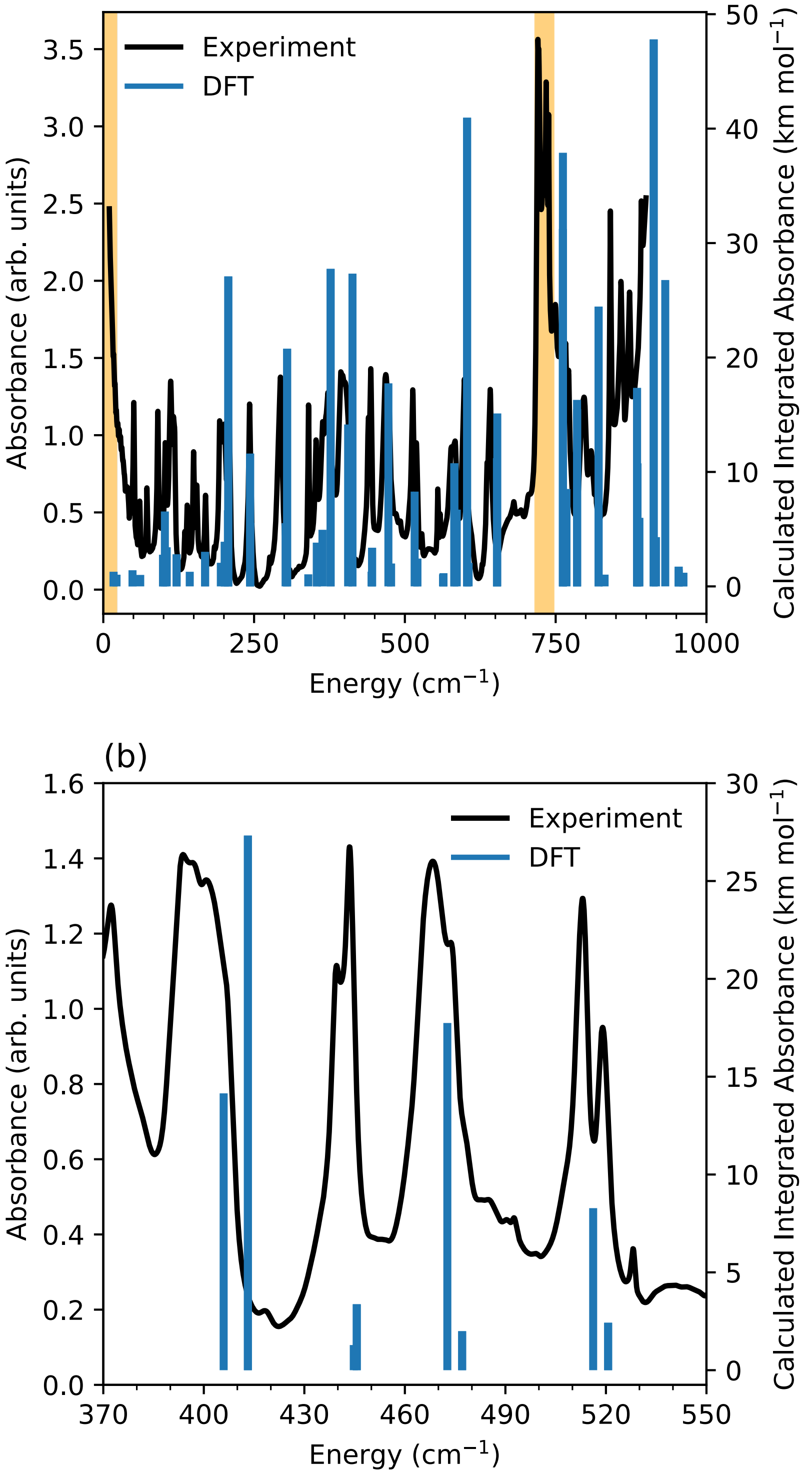

Supplementary Figure 2: (a) Experimental zero-field FTIR spectrum (black line) and *ab initio* DFT calculated infrared energies and intensities (blue lines) in the range 0 – 1000 cm^{-1}. Experimental spectra are plotted as the inverse of the transmission to highlight strong

vibrational modes. Artefacts at 0 and ~720 cm^{-1} (orange shaded regions) are caused by instrumental "blind spots" where the transmission is near zero because of destructive interference in the beam splitter employed in the FTIR spectrometer (b) Experimental zero-field FTIR spectrum (black line) and *ab initio* DFT calculated infrared energies and intensities (blue lines) in the range 370 – 550 cm^{-1}.

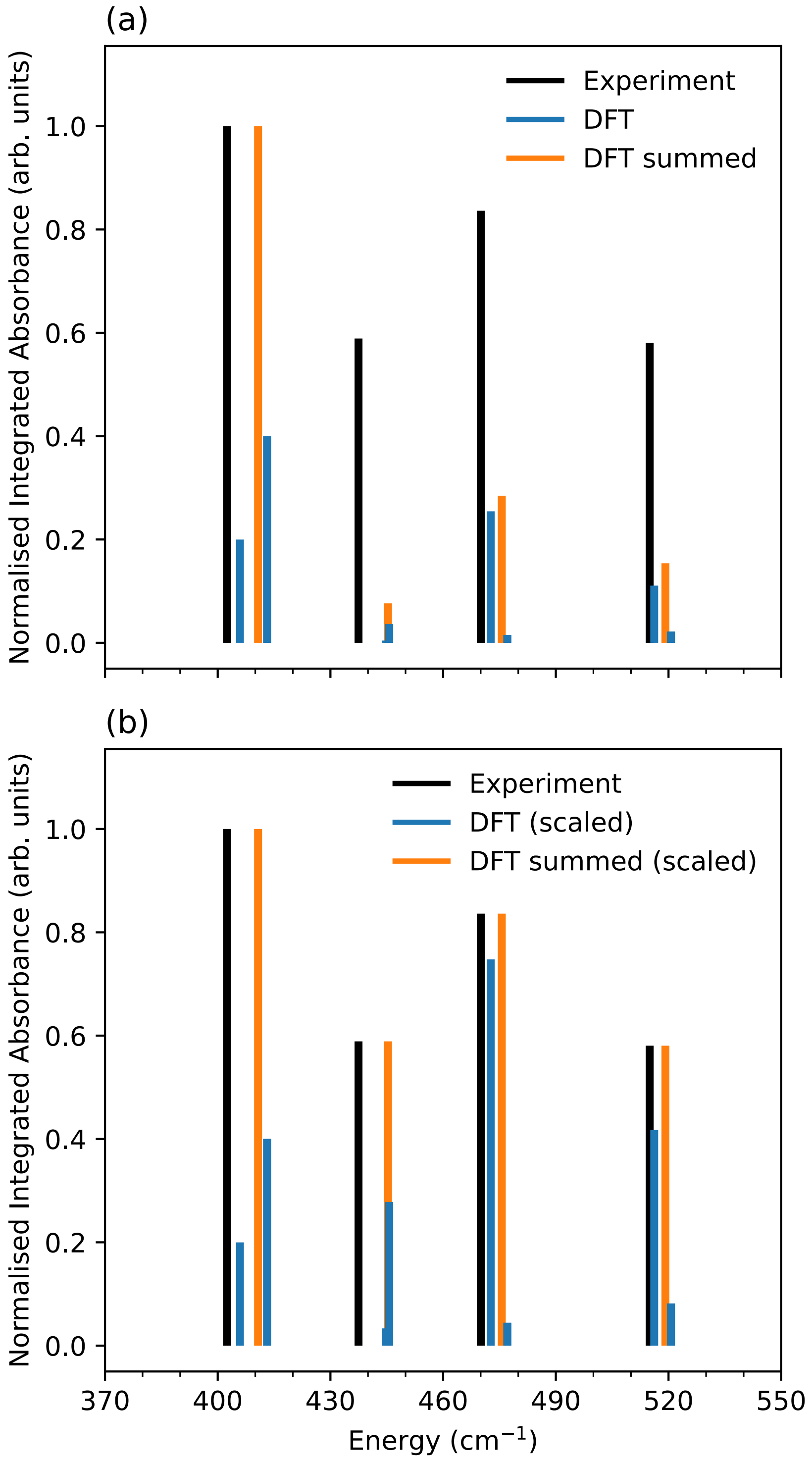

Supplementary Figure 3: (a) Experimental zero-field FTIR spectrum (black lines) and *ab initio* DFT calculated infrared energies and integrated absorbances (blue lines) in the range 370 – 550 cm^{-1}. Experimental transmission spectra are converted to absorption and then integrated within each of the four bands 385-420 cm^{-1}, 420-445 cm^{-1}, 455-485 cm^{-1}, 500-530 cm^{-1}, subsequently all bands are normalised such that the most intense band (385-420 cm^{-1}) has unit integrated absorbance. DFT calculated integrated absorbances are summed within the bands (orange lines) and again are normalised such that the sum of the most intense band (j = 34-36) has unit integrated absorbance. (b) DFT integrated absorbances scaled such that the sum of each band matches that of experiment, where the scaling factors are 7.71 (j = 37-39) 2.94 (j = 40-42), and 3.78 (j =43-45).

Supplementary Table 7: CASSCF-CASPT2-SO results for the $^2F_{7/2}$ ground term of $\mathbf{1_{opt}}$.

**Energy (cm^{-1})**	$\boldsymbol{g_\perp}$	$\boldsymbol{g_{\|\|}}$	**CF wave function composition**	$\langle \hat{J}_z \rangle$
0.0	2.62	5.12	70.974% $\|\pm 7/2\rangle$ + 16.329 %$\|\pm 1/2\rangle$ + 12.697 % $\|\mp 5/2\rangle$	±2.248
465.4	2.24	3.41	83.277 % $\|\pm 5/2\rangle$ + 16.533 % $\|\mp 7/2\rangle$ + 1.061 % $\|\mp 5/2\rangle$	±1.502
802.3	0.00	3.38	100% $\|\pm 3/2\rangle$	±1.500
1005.3	4.22	1.73	83.480 % $\|\pm 1/2\rangle$ + 12.493 % $\|\pm 7/2\rangle$ + 4.026 % $\|\mp 5/2\rangle$	±0.754

Supplementary Table 8: CASSCF-SO results for the $^2F_{7/2}$ ground term of $\mathbf{1_{opt}}$.

**Energy (cm^{-1})**	$g_{\perp}$	$g_{\|\|}$	**CF wave function composition**	$\langle \hat{J}_z \rangle$
0.0	2.40	5.55	74.792 % $\|\pm 7/2\rangle$ + 14.848 % $\|\pm 1/2\rangle$ + 10.360 % $\|\mp 5/2\rangle$	±2.433
320.0	2.09	3.69	85.098 % $\|\pm 5/2\rangle$ + 14.290 % $\|\mp 7/2\rangle$ + 0.613 % $\|\pm 1/2\rangle$	±1.624
607.0	0.00	3.39	100 % $\|\pm 3/2\rangle$	±1.500
723.2	4.28	1.59	84.539 % $\|\pm 1/2\rangle$ + 10.919 % $\|\pm 7/2\rangle$ + 4.542 % $\|\mp 5/2\rangle$	±0.691

Supplementary Table 9: CFPs from *ab initio* calculations on XRD and DFT-optimised geometries of **1**.

**CFP**	**1_{XRD} CASSCF-SO (cm^{-1})**	**1_{XRD} CASSCF-CASPT2-SO (cm^{-1})**	**1_{opt} CASSCF-SO (cm^{-1})**	**1_{opt} CASSCF-CASPT2-SO (cm^{-1})**
B_2^{-2}	-0.00001	-0.00017	0.00000	-0.00001
B_2^{-1}	0.00000	0.00068	-0.00001	-0.00018
B_2^{0}	-12.27600	-16.00657	-14.72160	-18.89621
B_2^{1}	-0.00002	0.00039	0.00001	-0.00020
B_2^{2}	0.00002	0.01249	0.00003	-0.00005
B_4^{-4}	0.00000	0.00007	0.00000	0.00004
B_4^{-3}	-2.28222	-0.82485	-4.67006	-6.70067
B_4^{-2}	0.00000	0.00000	0.00000	0.00000
B_4^{-1}	0.00000	-0.00002	0.00000	0.00000
B_4^{0}	0.01951	0.03373	0.03273	0.05452
B_4^{1}	0.00000	0.00020	0.00000	0.00001
B_4^{2}	0.00000	-0.00092	0.00000	0.00001
B_4^{3}	4.80565	-7.74786	-3.29563	-4.75338
B_4^{4}	0.00000	0.00015	0.00000	0.00000
B_6^{-6}	-0.04471	0.02025	0.06120	0.10212
B_6^{-5}	0.00000	0.00000	0.00000	0.00000
B_6^{-4}	0.00000	0.00000	0.00000	0.00000
B_6^{-3}	-0.01858	-0.01929	-0.05066	-0.07916
B_6^{-2}	0.00000	0.00000	0.00000	0.00000
B_6^{-1}	0.00000	0.00000	0.00000	0.00000
B_6^{0}	0.00588	0.00652	0.00599	0.00651
B_6^{1}	0.00000	-0.00001	0.00000	0.00000
B_6^{2}	0.00000	0.00006	0.00000	0.00000
B_6^{3}	0.05651	-0.09333	-0.03502	-0.05549
B_6^{4}	0.00000	0.00001	0.00000	0.00000
B_6^{5}	0.00000	0.00002	0.00000	0.00000
B_6^{6}	0.03662	0.09321	-0.01788	-0.03035

“Toy model” Hamiltonian

Calculating the populations of the lowest four states $|1_-,0\rangle, |1_-,1\rangle, |1_+,0\rangle$ and $|1_+,1\rangle$ as a function of magnetic field and vibrational mode energy reveals that, in the limit of $\hbar\omega \to 0$ and $B = 0$, all four states have equal population (Supplementary Figure 4a), but as the field strength is increased around 5 T ($\delta = 5$ cm^{-1}), $|1_-,0\rangle$ and $|1_-,1\rangle$ each have 50% population (Supplementary Figure 4b). Therefore, hot bands originating from excited electronic states will only be present at low fields (B < 5 T), irrespective of the vibrational mode energy. Alternatively, when the energy of the coupled vibrational mode increases above *ca.* 20 cm^{-1}, $|1_-,0\rangle$ and $|1_+,0\rangle$ each have 50% population in zero field (Supplementary Figure 4a), but when the field strength is increased to around 5 T, $|1_-,0\rangle$ approaches 100% population (Supplementary Figure 4b). Therefore, hot bands originating from an excited vibrational state will only be observable for low-energy vibrational modes ($\hbar\omega$ < 20 cm^{-1}).

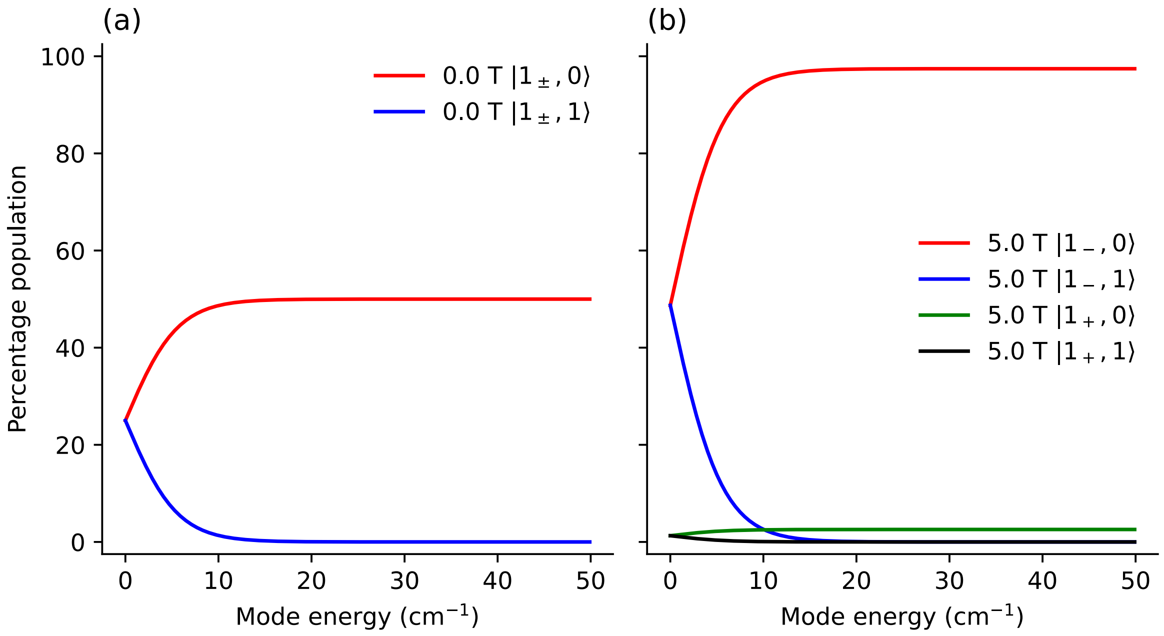

Supplementary Figure 4: Populations of the lowest four states of the simple vibronic model as a function of vibrational mode energy at 4.2 K in (a) zero applied magnetic field, (b) a 5 T magnetic field applied along the z axis.

The transition matrix elements due to the IR radiation between electronic states are herein defined as A_e and those between vibrational states as A_v. In the direct product basis $\{|1_-,0\rangle, |1_-,1\rangle, |1_+,0\rangle, |1_+,1\rangle, |2_-,0\rangle, |2_-,1\rangle, |2_+,0\rangle, |2_+,1\rangle\}$ the matrix form of the total transition moment, d_T, is:

$$
d_{\mathrm{T}} = \begin{bmatrix}
0 & A_{\mathrm{v}} & A_{\mathrm{e}} & 0 & A_{\mathrm{e}} & 0 & A_{\mathrm{e}} & 0 \\
A_{\mathrm{v}} & 0 & 0 & A_{\mathrm{e}} & 0 & A_{\mathrm{e}} & 0 & A_{\mathrm{e}} \\
A_{\mathrm{e}} & 0 & 0 & A_{\mathrm{v}} & A_{\mathrm{e}} & 0 & A_{\mathrm{e}} & 0 \\
0 & A_{\mathrm{e}} & A_{\mathrm{v}} & 0 & 0 & A_{\mathrm{e}} & 0 & A_{\mathrm{e}} \\
A_{\mathrm{e}} & 0 & A_{\mathrm{e}} & 0 & 0 & A_{\mathrm{v}} & A_{\mathrm{e}} & 0 \\
0 & A_{\mathrm{e}} & 0 & A_{\mathrm{e}} & A_{\mathrm{v}} & 0 & 0 & A_{\mathrm{e}} \\
A_{\mathrm{e}} & 0 & A_{\mathrm{e}} & 0 & A_{\mathrm{e}} & 0 & 0 & A_{\mathrm{v}} \\
0 & A_{\mathrm{e}} & 0 & A_{\mathrm{e}} & 0 & A_{\mathrm{e}} & A_{\mathrm{v}} & 0
\end{bmatrix} \qquad 1
$$

The vibronic coupling Hamiltonian $\widehat{H}_1$ is defined as below, as the direct product between the electronic and vibrational parts, where we have omitted the factor of $1/\sqrt{2}$ in the latter for simplicity (see Supplementary Equation 16). Diagonal terms G express energy shifts and off-diagonal terms F describe coupling between different electronic states.

$$
\widehat{H}_1 = \begin{bmatrix}
G & F & F & F \\
F & G & F & F \\
F & F & G & F \\
F & F & F & G
\end{bmatrix} \otimes \begin{bmatrix} 0 & 1 \\ 1 & 0 \end{bmatrix} = \begin{bmatrix}
0 & G & 0 & F & 0 & F & 0 & F \\
G & 0 & F & 0 & F & 0 & F & 0 \\
0 & F & 0 & G & 0 & F & 0 & F \\
F & 0 & G & 0 & F & 0 & F & 0 \\
0 & F & 0 & F & 0 & G & 0 & F \\
F & 0 & F & 0 & G & 0 & F & 0 \\
0 & F & 0 & F & 0 & F & 0 & G \\
F & 0 & F & 0 & F & 0 & G & 0
\end{bmatrix} \qquad 2
$$

Perturbation theory allows us to determine the first-order corrected eigenstates of $\widehat{H}_0 + \widehat{H}_1$ (main text Equation 1 plus Equation 2), yielding the un-normalised column eigenvectors below.

$$
\begin{matrix}
|1_-,0\rangle' & |1_-,1\rangle' & |1_+,0\rangle' & |1_+,1\rangle' & |2_-,0\rangle' & |2_-,1\rangle' & |2_+,0\rangle' & |2_+,1\rangle'
\end{matrix}
$$

$$
\begin{bmatrix}
1 & \frac{G}{\hbar\omega} & 0 & \frac{F}{\hbar\omega + 2\delta} & 0 & \frac{F}{\hbar\omega + \Delta} & 0 & \frac{F}{\hbar\omega + \Delta + 2\delta} \\
-\frac{G}{\hbar\omega} & 1 & -\frac{F}{\hbar\omega - 2\delta} & 0 & -\frac{F}{\hbar\omega - \Delta} & 0 & -\frac{F}{\hbar\omega - \Delta - 2\delta} & 0 \\
0 & \frac{F}{\hbar\omega - 2\delta} & 1 & \frac{G}{\hbar\omega} & 0 & \frac{F}{\hbar\omega + \Delta - 2\delta} & 0 & \frac{F}{\hbar\omega + \Delta} \\
-\frac{F}{\hbar\omega + 2\delta} & 0 & -\frac{G}{\hbar\omega} & 1 & -\frac{F}{\hbar\omega - \Delta + 2\delta} & 0 & -\frac{F}{\hbar\omega - \Delta} & 0 \\
0 & \frac{F}{\hbar\omega - \Delta} & 0 & \frac{F}{\hbar\omega - \Delta + 2\delta} & 1 & \frac{G}{\hbar\omega} & 0 & \frac{F}{\hbar\omega + 2\delta} \\
-\frac{F}{\hbar\omega + \Delta} & 0 & -\frac{F}{\hbar\omega + \Delta - 2\delta} & 0 & -\frac{G}{\hbar\omega} & 1 & -\frac{F}{\hbar\omega - 2\delta} & 0 \\
0 & \frac{F}{\hbar\omega - \Delta - 2\delta} & 0 & \frac{F}{\hbar\omega - \Delta} & 0 & \frac{F}{\hbar\omega - 2\delta} & 1 & \frac{G}{\hbar\omega} \\
-\frac{F}{\hbar\omega + \Delta + 2\delta} & 0 & -\frac{F}{\hbar\omega + \Delta} & 0 & -\frac{F}{\hbar\omega + 2\delta} & 0 & -\frac{G}{\hbar\omega} & 1
\end{bmatrix}
$$

3

Transition probabilities are proportional to the square of the off-diagonal matrix element of the transition moment (Supplementary Equation 2) between relevant perturbed states (*i.e.* transform Supplementary Equation 2 into the basis given by Supplementary Equation 3), and are given for the cold intra-KD excitations are given in the main text (Equation 3). Transition

probabilities for cold inter-KD vibronic excitations (absorption of an IR photon with $h\nu = \Delta + \hbar\omega$ and $h\nu = 2\delta + \Delta + \hbar\omega$) are given in Supplementary Equations 5 and 7, and their simplified forms under the approximations $\hbar\omega \ll \Delta$ and $\delta \ll \Delta$, are given in Supplementary Equations 6 and 8, respectively; these expressions diverge when $\delta \to 0$ and/or $\hbar\omega \to 0$ (*i.e.* in proximity to the purely electronic inter-KD transitions).

$$I(|1_-,0\rangle \to |1_+,1\rangle) \propto \left(F\left(A_\mathrm{e}\left(\frac{1}{\hbar\omega - \Delta} - \frac{1}{\hbar\omega + \Delta} + \frac{1}{\hbar\omega + 2\delta - \Delta} - \frac{1}{\Delta + \hbar\omega + 2\delta}\right) + A_\mathrm{v}\left(\frac{F}{(\Delta + \hbar\omega + 2\delta)(\Delta - \hbar\omega)} - \frac{F}{(2\delta - \Delta + \hbar\omega)(\Delta + \hbar\omega)} - \frac{2G}{2\delta\hbar\omega + \hbar^2\omega^2}\right)\right)\right)^2 \quad 4$$

$$I(|1_-,0\rangle \to |2_-,1\rangle) \propto \left(F\left(A_\mathrm{e}\left(\frac{1}{\hbar\omega - 2\delta} - \frac{1}{\hbar\omega + 2\delta} + \frac{1}{\Delta + \hbar\omega - 2\delta} - \frac{1}{\Delta + \hbar\omega + 2\delta}\right) + A_\mathrm{v}\left(\frac{F}{(\Delta + \hbar\omega + 2\delta)(2\delta - \hbar\omega)} - \frac{F}{(\Delta + \hbar\omega - 2\delta)(2\delta + \hbar\omega)} - \frac{2G}{\hbar\omega(\Delta + \hbar\omega)}\right)\right)\right)^2 \quad 5$$

$$I(|1_-,0\rangle \to |2_-,1\rangle) \propto \left(F\left(A_\mathrm{e}\left(\frac{1}{\hbar\omega - 2\delta} - \frac{1}{\hbar\omega + 2\delta}\right) + A_\mathrm{v}\left(\frac{F}{\Delta(2\delta - \hbar\omega)} - \frac{F}{\Delta(2\delta + \hbar\omega)} - \frac{2G}{\Delta\hbar\omega}\right)\right)\right)^2 \quad 6$$

$$I(|1_-,0\rangle \to |2_+,1\rangle) \propto \left(2A_\mathrm{v}F\left(\frac{F}{(\Delta + \hbar\omega)(2\delta + \hbar\omega)} + \frac{G}{\hbar\omega(2\delta + \hbar\omega + \Delta)}\right)\right)^2 \quad 7$$

$$I(|1_-,0\rangle \to |2_+,1\rangle) \propto \left(2A_\mathrm{v}F\left(\frac{F}{\Delta(2\delta + \hbar\omega)} + \frac{G}{\Delta\hbar\omega}\right)\right)^2 \quad 8$$

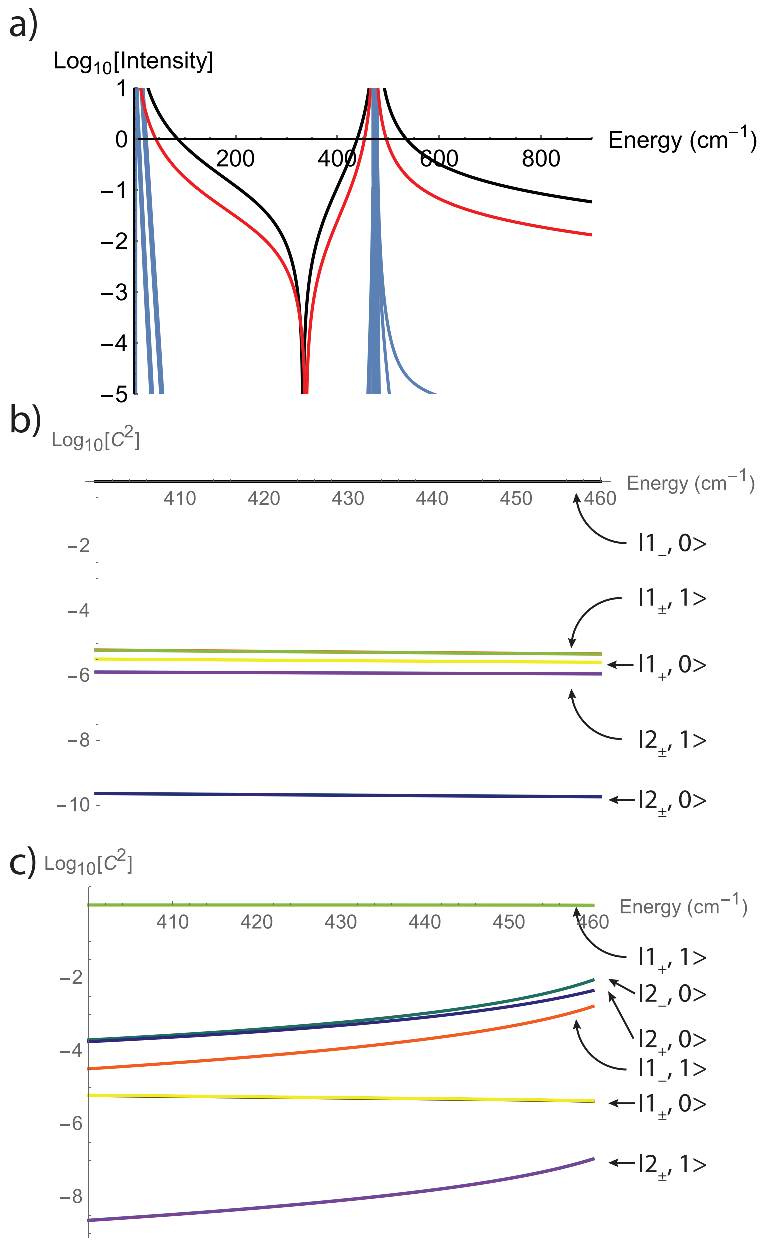

Supplementary Figure 5: a) Absorption intensity (including Boltzmann population of the initial states at 4 K) for all possible transitions in the total toy-model of $\hat{H}_0 + \hat{H}_1$ (Equations 1 and 2), under irradiation from an IR source with uniform intensity and uniform vibronic coupling, calculated by exact diagonalisation. Electronically cold intra-KD vibronic transitions ($|1_-, 0\rangle' \rightarrow |1_+, 1\rangle'$) are shown in black and electronically hot intra-KD vibronic transitions ($|1_+, 0\rangle' \rightarrow |1_-, 1\rangle'$) are shown in red, however such assignments fail near divergences; all other states in blue. Constructed with model parameters F = G = 1 cm^{-1}, A_{v} = 10^2, A_{e} = 1, Δ = 474 cm^{-1} and δ = 2 cm^{-1} (field *ca.* 2 T). b) and c) Squared eigenvector coefficients for the initial state $|1_-, 0\rangle'$ (b) and the final state $|1_+, 1\rangle'$ (c) involved in the cold intra-KD vibronic transition, calculated by exact diagonalisation.

Ab initio vibronic coupling and simulation of FIRMS maps

When constructing the total *ab initio* vibronic coupling Hamiltonian, $\hat{H}_{\mathrm{T}}$ (Equation 4), the electronic Hamiltonians in the $|m_J\rangle$ basis ($\hat{H}_{\mathrm{CF}} + \hat{H}_{\mathrm{Zee}}$) can be transformed into the direct product basis $|m_J, n_1, n_2, \ldots\rangle$ by taking the Kronecker product with an identity matrix the size of the vibrational basis ($(n_{\mathrm{max}} + 1)^{n_{\mathrm{vib}}}$, where n_{max} is the maximum number of vibrational quanta considered, herein $n_{\mathrm{max}} = 1$, and n_{vib} is the number of vibrational modes considered). Likewise, each vibrational matrix ($\hat{H}_{\mathrm{vib},j}$, constructed in its own vibrational basis) can be transformed into the direct product basis by taking the Kronecker product with an identity matrix the size of the electronic basis ($2J + 1 = 8$) and for each of the other vibrational modes, taking careful consideration of the ordering of the basis; this is summarised in Supplementary Equation 9:

$$\begin{aligned}\hat{H}_{\mathrm{T}} = {} & \left(\hat{H}_{\mathrm{Zee}} + \hat{H}_{\mathrm{CF}}\right) \otimes \mathbb{I}_{\mathrm{vib},1} \otimes \mathbb{I}_{\mathrm{vib},2} \otimes \ldots + \mathbb{I}_{\mathrm{elec}} \otimes \hat{H}_{\mathrm{vib},1} \otimes \mathbb{I}_{\mathrm{vib},2} \\ & \otimes \ldots + \hat{H}_{\mathrm{coup-e},1} \otimes \hat{H}_{\mathrm{coup-v},1} \otimes \mathbb{I}_{\mathrm{vib},2} \otimes \ldots + \mathbb{I}_{\mathrm{elec}} \\ & \otimes \mathbb{I}_{\mathrm{vib},1} \otimes \hat{H}_{\mathrm{vib},2} \otimes \ldots + \hat{H}_{\mathrm{coup-e},2} \otimes \mathbb{I}_{\mathrm{vib},1} \otimes \hat{H}_{\mathrm{coup-v},2} \\ & \otimes \ldots + \ldots\end{aligned} \qquad 9$$

To obtain $\left(\partial B_k^q / \partial Q_j\right)_{\mathrm{eq}}$ for each vibrational mode in Equation 9, we distort the molecular geometry along the normal mode coordinate up to some maximum displacement (*vide infra*) and calculate the electronic structure with CASSCF-SO (differences in CFPs are taken from the electronic states calculated at equilibrium using CASSCF-SO, Supplementary Table 7). Assuming the harmonic approximation for each vibrational mode, we define the maximal cartesian displacement vector of a given mode j in its nth harmonic state as:

$$\vec{Q}_{j,n} = \sqrt{\frac{\hbar\left(2n_j + 1\right)}{\omega_j \mu_j}}\,\hat{\vec{Q}}_j \qquad 10$$

Where μ_j is the reduced mass of mode j, and $\hat{\vec{Q}}_j$ is a unit vector containing each atomic displacement of mode j. We define the zero-point displacement (ZPD) of a mode as the magnitude of Supplementary Equation 10 when n_j = 0:

$$Q_{j,\mathrm{ZPD}} = \left|\vec{Q}_{j,0}\right| = \sqrt{\frac{\hbar}{\omega_j \mu_j}} \qquad 11$$

Simply setting the maximal displacement of each mode equal to its ZPD does not accurately capture the fact that, even at low temperatures, there is a non-zero population of excited

vibrational states which have larger displacements. Therefore, we calculate the thermal population of excited states using the Boltzmann distribution and thus determine the thermally averaged displacement for each mode at some specific temperature. In this work, we choose a temperature of 4 K which corresponds roughly to that of the experimental FIRMS spectrum and displace the molecule up to ±1.5× the zero-point displacement (ZPD) for all modes.

When distorting along each mode, we calculate the electronic structure with CASSCF-SO at 4 evenly spaced points in both positive and negative directions up to ±1.5× $Q_{j,\mathrm{ZPD}}$, and fit each crystal field parameter at these points to a cubic polynomial (Supplementary Figure 6, Supplementary Equation 12, where ${B_k^q}_{\mathrm{eq}}$ is the set of CFPs obtained from the optimised structure using CASSCF-SO). Here, Q_j is the dimensionless fraction of $Q_{j,\mathrm{ZPD}}$, thus Q_j = 1 corresponds to a physical displacement of the atoms along $\hat{\vec{Q}}_j$ up to $Q_{j,\mathrm{ZPD}}$ (Supplementary Equations 10 and 11).

$$B_k^q(Q_j) = aQ_j^3 + bQ_j^2 + cQ_j + {B_k^q}_{\mathrm{eq}} \qquad 12$$

Defining the equilibrium geometry as $Q_j = 0$ the first derivative of Equation S12 with respect to Q_j evaluated at equilibrium is simply the linear coefficient: $\left(\frac{\partial B_k^q}{\partial Q_j}\right)_{\mathrm{eq}} = c$. Working still in our ZPD based unit system, we define Q_j as the harmonic oscillator position operator using the raising and lowering operators|

$$Q_j = \frac{1}{\sqrt{2}}\left(\hat{a}^\dagger + \hat{a}\right) \qquad 13$$

Which is evaluated in the basis of harmonic eigenstates yielding non-zero matrix elements of the kind

$$\langle n_j - 1|Q_j|n_j\rangle = \frac{1}{\sqrt{2}}\langle n_j - 1|\hat{a}|n_j\rangle = \sqrt{\frac{n_j}{2}} \qquad 14$$

$$\langle n_j + 1|Q_j|n_j\rangle = \frac{1}{\sqrt{2}}\langle n_j + 1|\hat{a}^\dagger|n_j\rangle = \sqrt{\frac{n_j + 1}{2}} \qquad 15$$

As we restrict ourselves to the $n_j = 0, 1$ basis, the vibrational part becomes:

$$\hat{H}_{\mathrm{coup-v},j} = \begin{bmatrix} 0 & \frac{1}{\sqrt{2}} \\ \frac{1}{\sqrt{2}} & 0 \end{bmatrix} \qquad 16$$

To calculate the electric dipole moment matrix elements, we first define the electric dipole moment μ_{E} for a single particle between a pair of states (where z is the particle's charge, and r is the position operator):

$$\mu_{\mathrm{E}_{fi}} = \langle \psi_f | zr | \psi_i \rangle \tag{17}$$

For a system of many particles this becomes:

$$\mu_{\mathrm{E}_{fi}} = \langle \psi_f | z_1 r_1 + z_2 r_2 + \cdots | \psi_i \rangle \tag{18}$$

In our case, we calculate the electric dipole moment in the basis of harmonic vibrational states $|n\rangle$. This is typically accomplished by expanding the electric dipole moment as a Taylor series in vibrational mode coordinate Q, such that for mode j:[2]

$$\mu_{\mathrm{E}} = \mu_{\mathrm{E}}^{(0)} + \left(\frac{\partial \mu_{\mathrm{E}}}{\partial Q_j}\right)_{\mathrm{eq}} Q_j + \frac{1}{2}\left(\frac{\partial^2 \mu_{\mathrm{E}}}{\partial Q_j^2}\right)_{\mathrm{eq}} Q_j^2 + \cdots \tag{19}$$

The first term is independent of Q and is simply the electric dipole moment of the system at equilibrium (*i.e.* the permanent electric dipole) and so can be ignored. The term linear in Q is the transition dipole moment for a single quantum transition (*e.g.* $|0\rangle \rightarrow |1\rangle$), while higher order terms correspond to overtones (*e.g.* $|0\rangle \rightarrow |2\rangle$), which are small by comparison and are therefore excluded. Then, the electric transition dipole moment matrix elements are:

$$\langle n_j' | \mu_{\mathrm{E}} | n_j \rangle = \left\langle n_j' \middle| \left(\frac{\partial \mu_{\mathrm{E}}}{\partial Q_j}\right)_{\mathrm{eq}} Q_j \middle| n_j \right\rangle = \left(\frac{\partial \mu_{\mathrm{E}}}{\partial Q_j}\right)_{\mathrm{eq}} \langle n_j' | Q_j | n_j \rangle \tag{20}$$

The term $\left(\frac{\partial \mu_{\mathrm{E}}}{\partial Q_j}\right)_{\mathrm{eq}}$ is the derivative of the electric dipole moment with respect to the vibrational mode coordinate. We use the results of our DFT optimisation to obtain this derivative for each mode, however Gaussian does not use our ZPD based coordinate system, and instead uses the "textbook" definition of the harmonic oscillator displacement operator which we refer to as $\tilde{Q}_j$ and note that the two definitions are related by

$$\tilde{Q}_j = Q_{j,\mathrm{ZPD}} \cdot Q_j \tag{21}$$

Therefore, to match the value of $\left(\frac{\partial \mu_{\mathrm{E}}}{\partial \tilde{Q}_j}\right)_0$ calculated by Gaussian, when evaluating $\tilde{Q}$ in the harmonic basis we use:

$$\tilde{Q}_j = Q_{j,\mathrm{ZPD}} \cdot Q_j = \sqrt{\frac{\hbar}{\mu_j \omega_j}} \cdot \frac{1}{\sqrt{2}} \left(a^{\dagger} + a\right) = \sqrt{\frac{\hbar}{4\pi c \bar{\nu}_j \mu_j}} \left(a^{\dagger} + a\right) \tag{22}$$

$$a^{\dagger}|n\rangle = \sqrt{n+1}|n+1\rangle \qquad 23$$

$$a|n\rangle = \sqrt{n}|n-1\rangle$$

Which gives the non-zero matrix elements:

$$\langle n_j - 1|\tilde{Q}_j|n_j\rangle = \sqrt{\frac{\hbar}{2\mu_j\omega_j}}\langle n_j - 1|\hat{a}|n_j\rangle = \sqrt{\frac{\hbar n_j}{2\mu_j\omega_j}} \qquad 24$$

$$\langle n_j + 1|\tilde{Q}_j|n_j\rangle = \sqrt{\frac{\hbar}{2\mu_j\omega_j}}\langle n_j + 1|\hat{a}^{\dagger}|n_j\rangle = \sqrt{\frac{\hbar(n_j+1)}{2\mu_j\omega_j}} \qquad 25$$

Therefore, in terms of $\tilde{Q}_j$

$$\langle n_j'|\mu_{\mathrm{E}}|n_j\rangle = \left(\frac{\partial\mu_{\mathrm{E}}}{\partial\tilde{Q}_j}\right)_{\mathrm{eq}}\langle n_j'|\tilde{Q}_j|n_j\rangle \qquad 26$$

$j = 4$

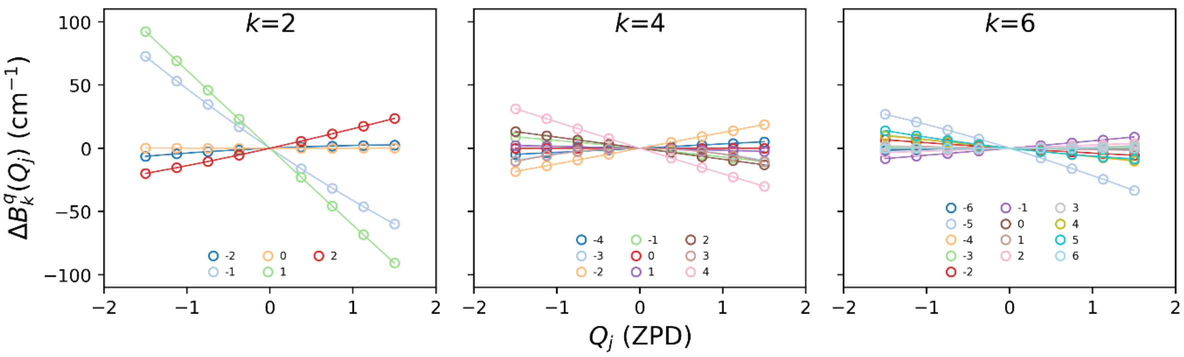

$j = 5$

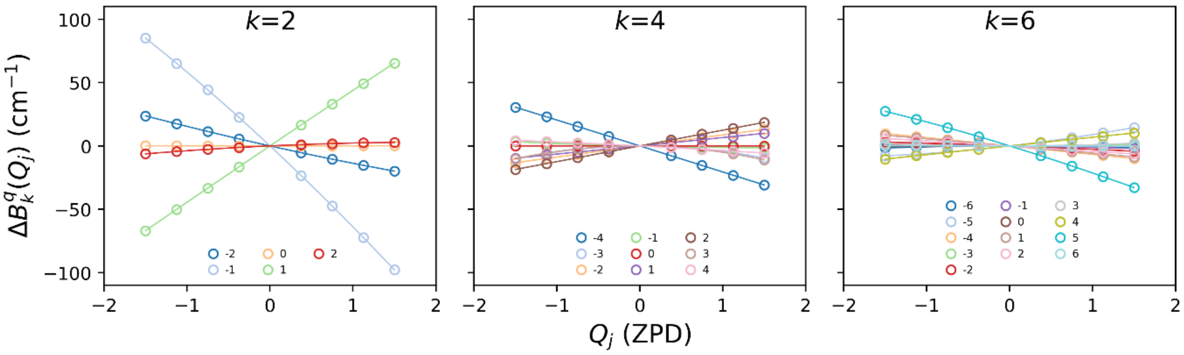

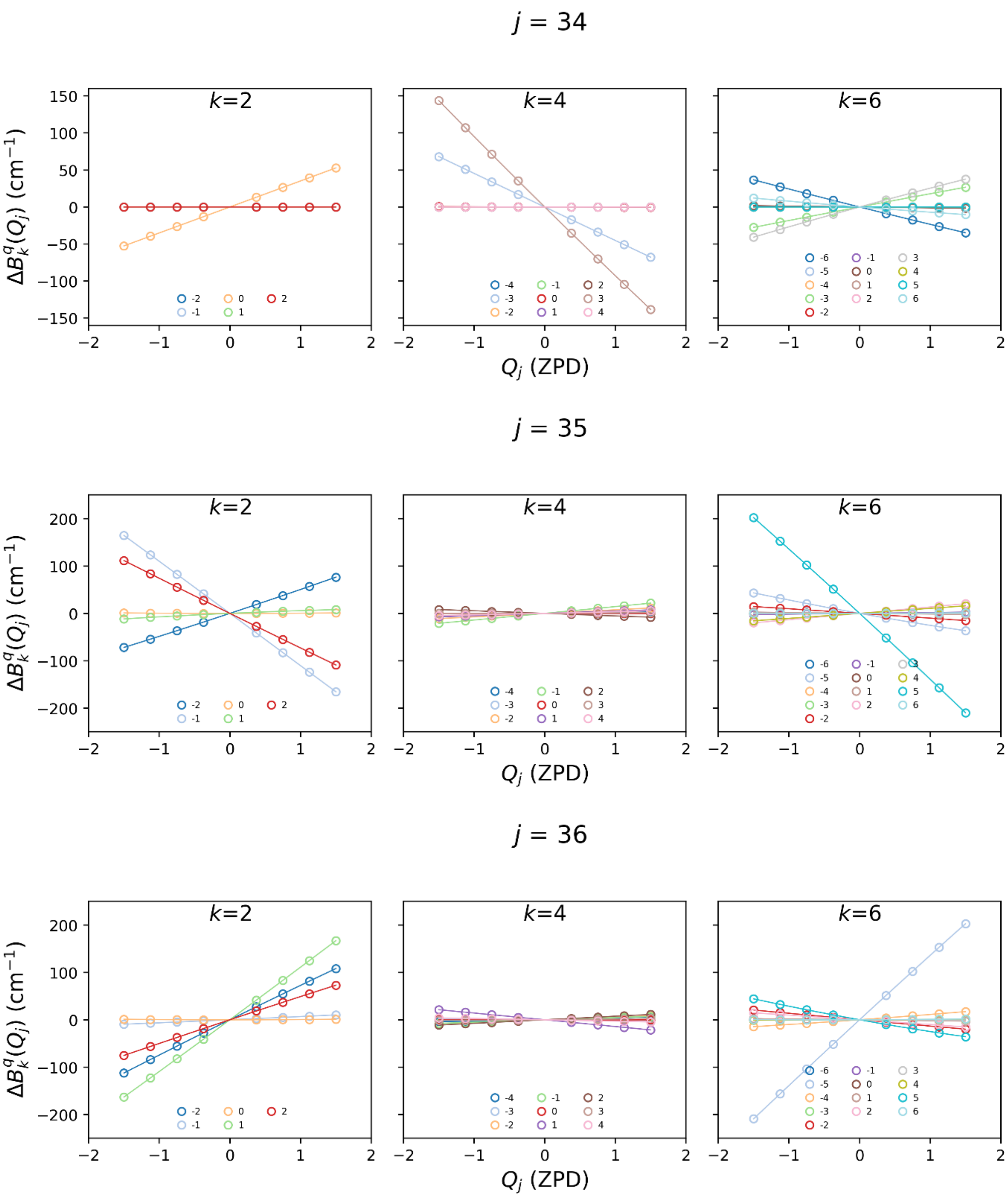
j = 34
j = 35
j = 36
k=2
k=4
k=6
ΔB_k^q(Q_j) (cm^-1)
Q_j (ZPD)
150
100
50
0
−50
−100
−150
200
−200
−2
−1
1
2
-6
-5
-4
-3
-2
-1
0
1
2
3
4
5
6

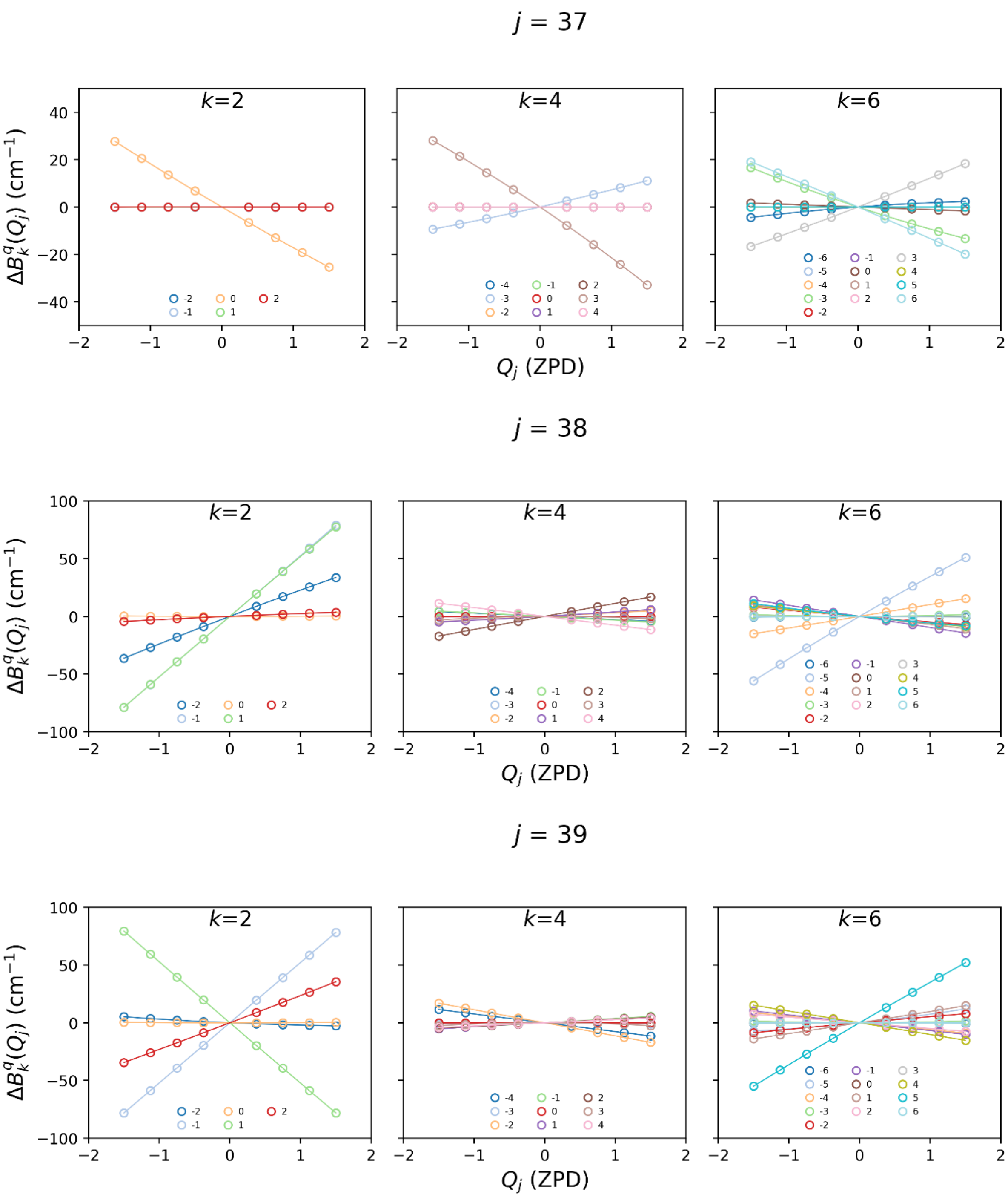

j = 37
k=2
k=4
k=6
ΔB_k^q(Q_j) (cm⁻¹)
Q_j (ZPD)
j = 38
k=2
k=4
k=6
ΔB_k^q(Q_j) (cm⁻¹)
Q_j (ZPD)
j = 39
k=2
k=4
k=6
ΔB_k^q(Q_j) (cm⁻¹)
Q_j (ZPD)

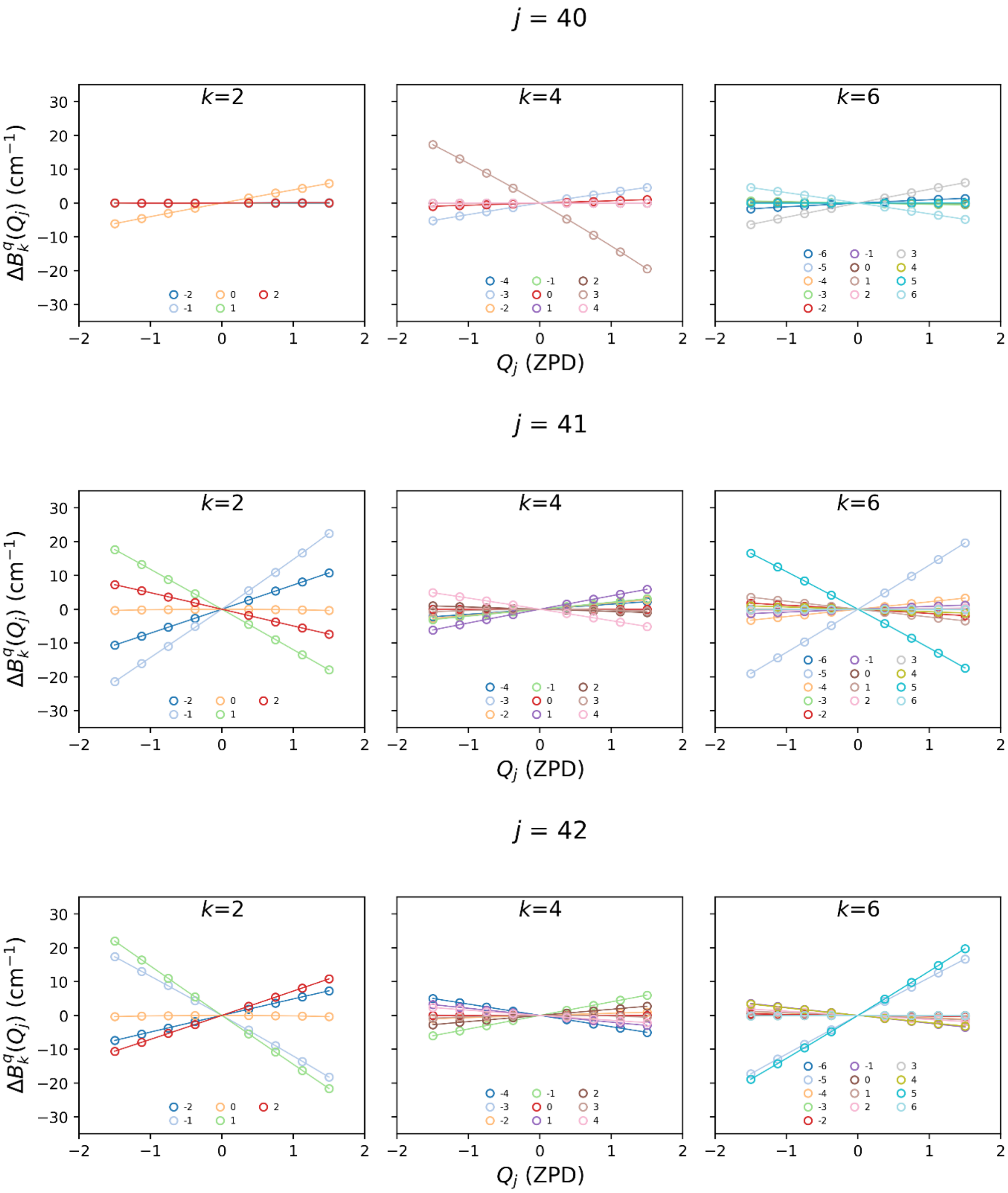
j = 40
j = 41
j = 42
k=2
k=4
k=6
ΔB_k^q(Q_j) (cm^−1)
Q_j (ZPD)
-2 -1 0 1 2
-4 -3 -2 -1 0 1 2 3 4
-6 -5 -4 -3 -2 -1 0 1 2 3 4 5 6

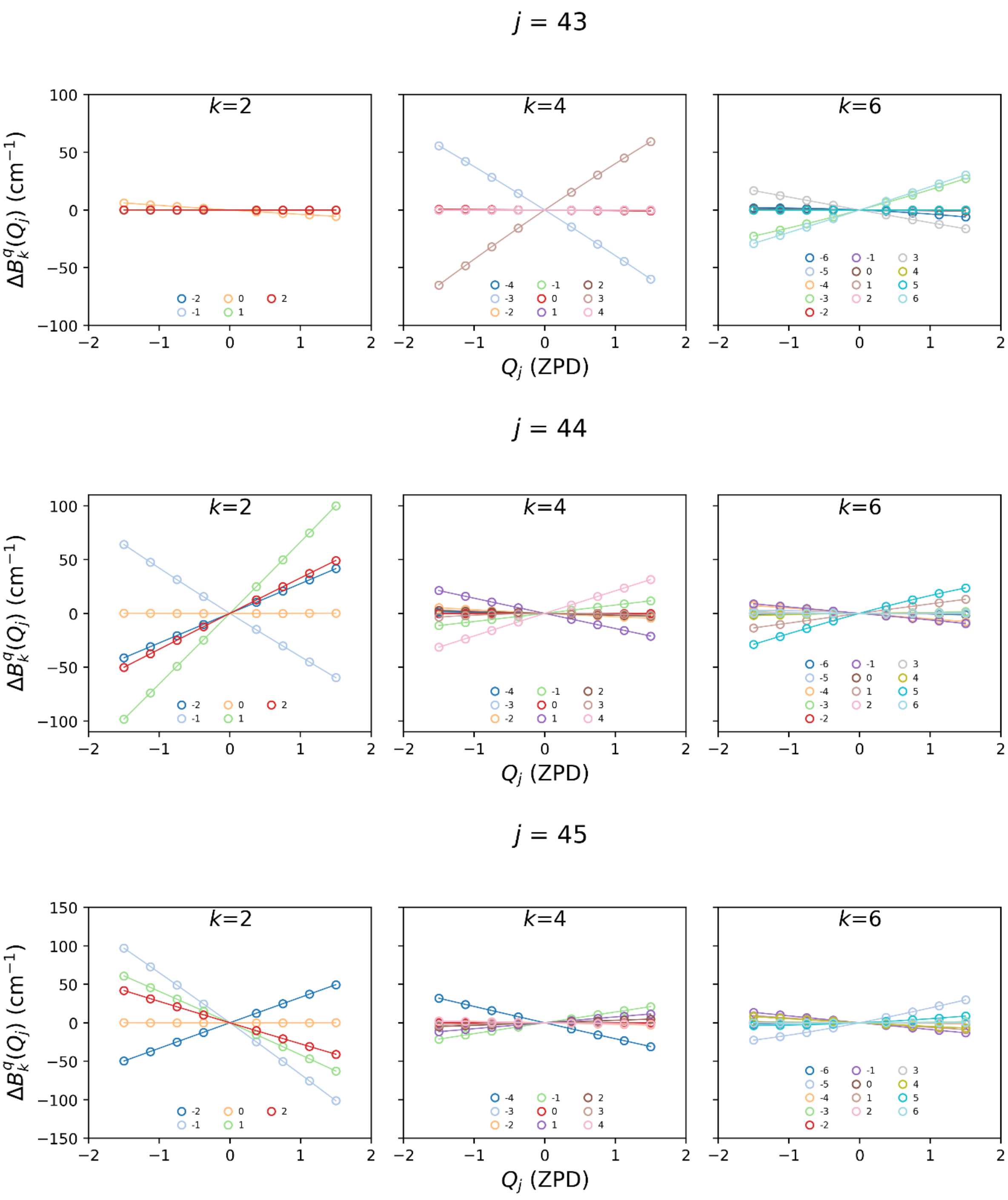

Supplementary Figure 6: Fits of the changes in the crystal field parameters with third order polynomials as a function of displacement along vibrational modes 4, 5, 34-45. All fits are clearly dominated by linear terms, justifying our first-order Taylor expansion.

Experimental and theoretical FIRMS maps and analysis

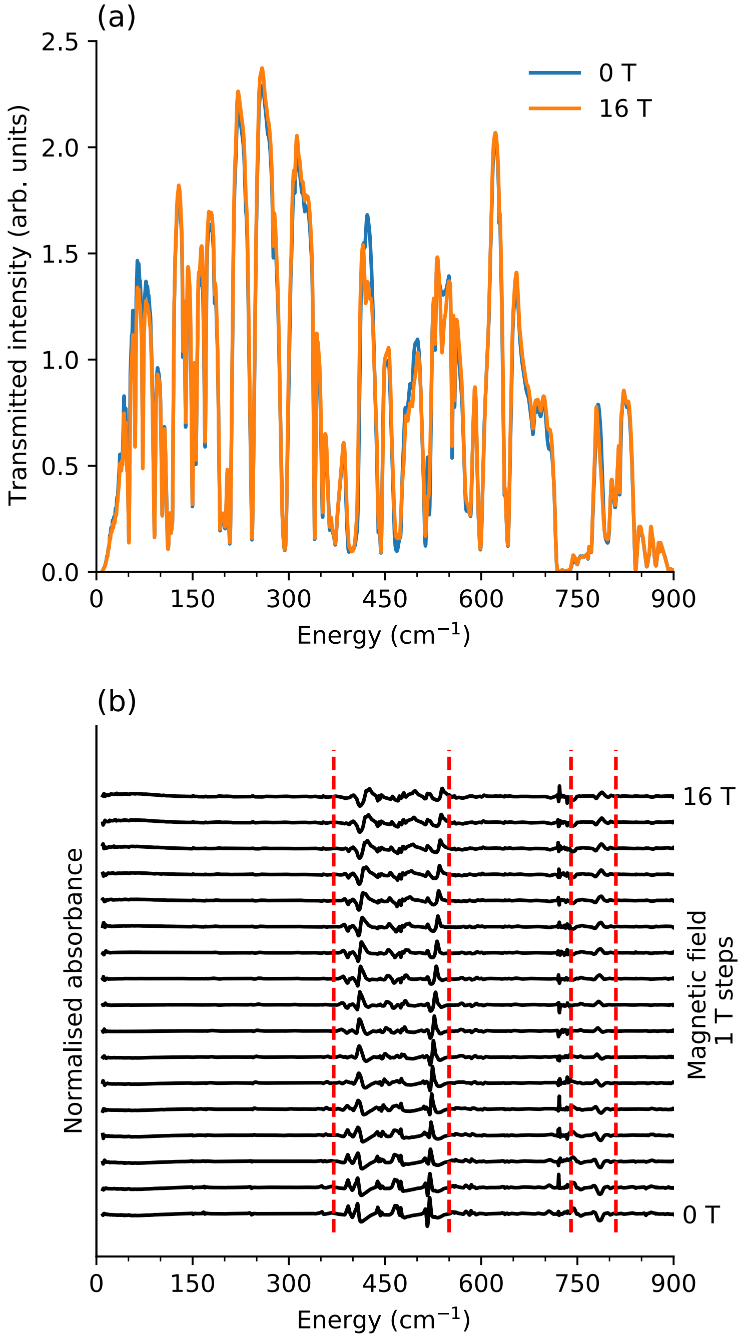

Supplementary Figure 7: Far-infrared magnetospectroscopy (FIRMS) data for [Yb(trensal)]. (a) Raw FTIR spectra taken in 0 T and 16 T applied magnetic fields. (b) FIRMS spectra at different applied magnetic fields, normalised by the average of all spectra. Intense field dependent spectral features are highlighted by this division and appear in the ranges 370 to 550 cm^{-1} and 740 to 815 cm^{-1} (between dashed red lines – the features just below 740 cm^{-1}

are artifacts caused by the spectrometer blind spot at ~720 cm^{-1}). All measurements were performed at 4.2 K.

A brief summary of the assignment of the main features of the FIRMS map is given in the main text, but here we give a more detailed account.

We begin with signal **A** ($E_{B=0}$ = 393 cm^{-1}) which moves to lower energy with increasing field; this is consistent with an electronically hot intra-KD transition $|1_+, 0\rangle \rightarrow |1_-, 1\rangle$. This signal intersects with a band moving to higher energy in the field range 8 – 10 T and therefore could show some avoided crossing type behaviour; unfortunately, due to the low intensity of signal **A**, such behaviour is not seen within our current data. The nearby signal **B** ($E_{B=0}$ = 407 cm^{-1}) is likely the same as side-band 2a in the luminescence data (Figure 2), and is consistent with a cold intra-KD vibronic transition $|1_-, 0\rangle \rightarrow |1_+, 1\rangle$. Both **A** and **B** emanate from a region of low IR transmittance that shows only small shifts at its edges due to vibronic coupling, hence their apparent field-independence near zero-field is likely an artefact of the normalisation scheme. Our DFT calculations predict a vibrational mode at 406 cm^{-1} (mode 34, A symmetry) which involves compression of the entire structure along the axial Yb-NR_3 direction (Supplementary Video 13), and a pair of modes of E symmetry at 413 cm^{-1} (modes 35 and 36) which are anti-symmetric N-Yb-N stretches (Supplementary Table 5, Supplementary Movies 14 and 15). Considering the excellent agreement with the zero-field FTIR spectrum (Supplementary Figure 2) and that there are no other vibrational modes within ±25 cm^{-1} of the **A** and **B** signals, we are confident with these assignments. Simulations of FIRMS maps obtained from coupling to modes 34-36 simultaneously (Supplementary Figure 7) gives further weight to our assignment, allowing us to reproduce the broad nature of the vibronic signals seen in the experiment, specifically the dominant band at ~410 cm^{-1} that moves to higher energy with increasing field.

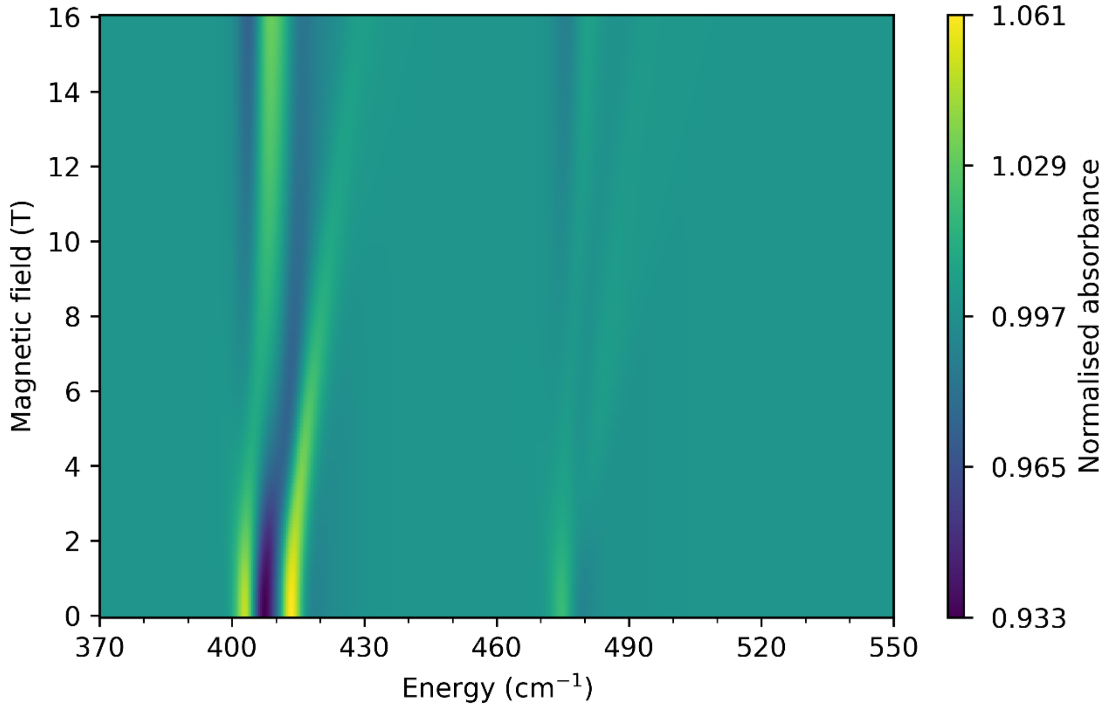

Supplementary Figure 8: Simulated FIRMS map coupling to modes 34-36 using CASSCF-CASPT2-SO equilibrium CFPs, CASSCF-SO spin-phonon couplings, and *ab initio* transition intensities.

Signal **C** ($E_{B=0}$ = 444 cm^{-1}) consists of an electronically hot intra-KD band which disappears at ~ 6 T and a cold intra-KD band. Our DFT results indicate a mode at 444 cm^{-1} (mode 37, A) in which the structure is compressed along the Yb-N dative bond (Supplementary Video 16), and a pair of modes at 446 cm^{-1} (modes 38 and 39, E) which are similar to modes 35 and 36 but include considerable motion of the amine nitrogen (Supplementary Videos 17 and 18). Simulations of the FIRMS map agree very well with the experimental data (Supplementary Figure 8), specifically the weaker overall intensity compared to features **A** and **B** and the very broad high field arm around 450 – 460 cm^{-1}.

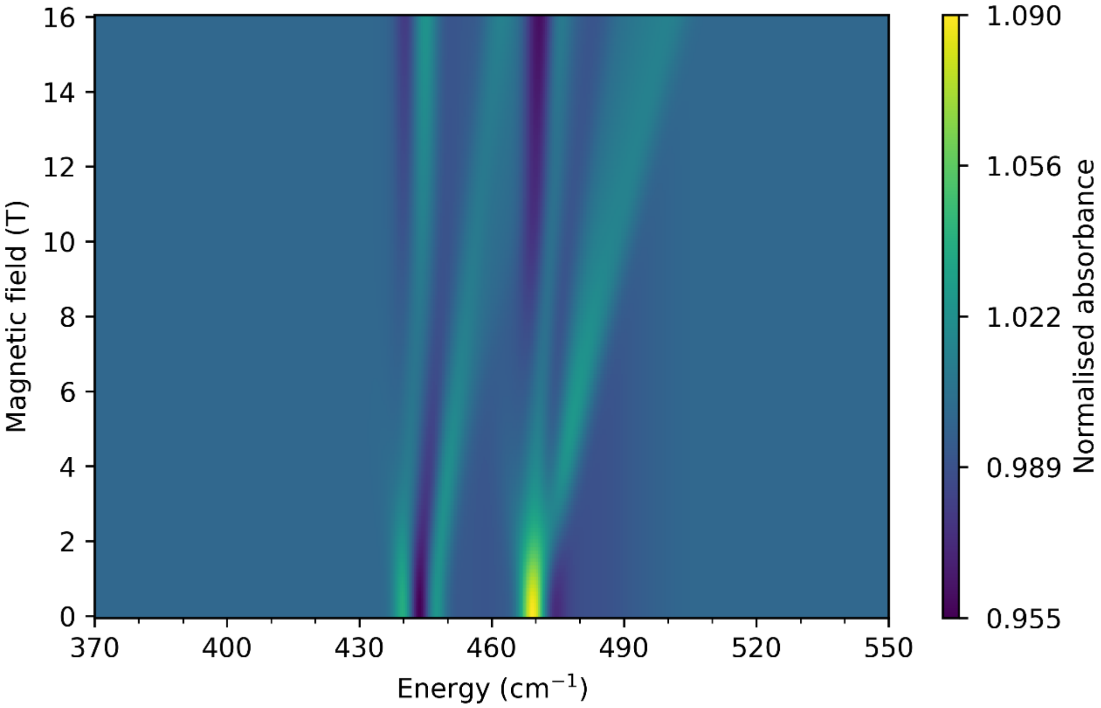

Supplementary Figure 9: Simulated FIRMS map coupling to modes 37-39 using CASSCF-CASPT2-SO equilibrium CFPs, CASSCF-SO spin-phonon couplings, and *ab initio* transition intensities.

Signals **D** ($E_{B=0}$ = 468 cm^{-1}) and **E** ($E_{B=0}$ = 474 cm^{-1}) are composed of many features at zero field which are poorly resolved, though we clearly see both hot and cold electronic bands moving as a function of field. Further, we note that signal **D** intersects with a band from signal **C** giving the appearance of an avoided crossing, but, similar to signal **A**, we do not see any interaction between the two, perhaps due to the low intensity of the peak that moves to low energy with field. Signals **D** and **E** are complicated as they are very close to the purely electronic transition at 474 cm^{-1}, and, hence, could be electronic ($|1_{\pm},n\rangle \rightarrow |2_{\pm},n\rangle$), intra-KD cold ($|1_{\pm},0\rangle \rightarrow |1_{\pm},1\rangle$, $\hbar\omega$ ~ 470 cm^{-1}) or inter-KD hot ($|1_{\pm},0\rangle \rightarrow |2_{\pm},1\rangle$, $\hbar\omega$ < 20 cm^{-1}) bands. Our DFT calculations do not clarify matters, as they reveal a mode at 473 cm^{-1} (mode 40, A, out-of-plane ring deformation of all aromatic rings, Supplementary Video 19), and two modes at 477 cm^{-1} (modes 41, 42, E, out-of-phase equivalents of mode 40, Supplementary Videos 20 and 21). While in reality there will be numerous acoustic phonon modes below 20 cm^{-1}, the IR transition intensities for these modes will be negligible compared to intramolecular modes. However, we cannot rule out contributions from hot vibrational bands. Nonetheless, our simulations of these signals using modes 40-42 are good (Supplementary Figure 9), showing the broad band of intensity at zero field and overall very weak intensity.

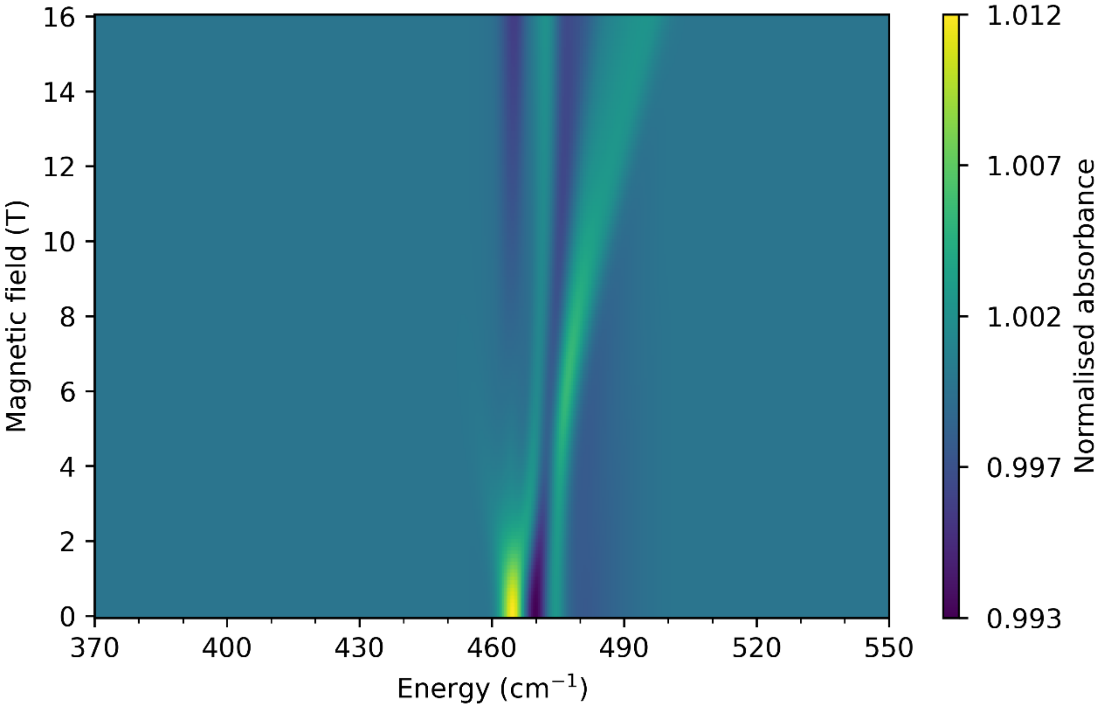

Supplementary Figure 10: Simulated FIRMS map coupling to modes 40-42 using CASSCF-CASPT2-SO equilibrium CFPs, CASSCF-SO spin-phonon couplings, and *ab initio* transition intensities.

The very intense signal **F** ($E_{B=0}$ = 520 cm^{-1}) corresponds to peak 2b in the luminescence measurements (Figure 2), and appears as a weak electronic hot band which fades for B > 5 T and a much stronger cold band. As these are higher in energy than the purely electronic transition, they could be either intra- or inter-KD vibronic transitions or both. In the case of an intra-KD transition, our DFT results indicate a mode of A symmetry at 516 cm^{-1} (mode 43) and a pair of modes of E symmetry at 521 cm^{-1} (modes 44 and 45), all of which are stretches of each N-Yb-O unit (Supplementary Videos 22-24). Alternatively, if these were inter-KD transitions, the energy of the vibrational mode would have to be *ca.* 46 cm^{-1}: there are indeed a pair of modes at 48 cm^{-1} (modes 4 and 5, E) which involve a rocking motion of the entire trensal unit (Supplementary Videos 1 and 2). Simulation of a FIRMS map including modes 43, 44 and 45 suggests a slightly richer signal than that seen in experiment, whereas a simulation including modes 4 and 5 gives no signal at all. Although neither of these observations are perfectly in agreement with the experiment (Figure 3), coupling to modes 43-45 does yield filed dependent signal at the right energy. Thus, we suggest that signal **F** is a cold intra-KD transition $|1_{\pm},0\rangle \rightarrow |1_{\mp},1\rangle$ coupling to vibrational modes 43-45.

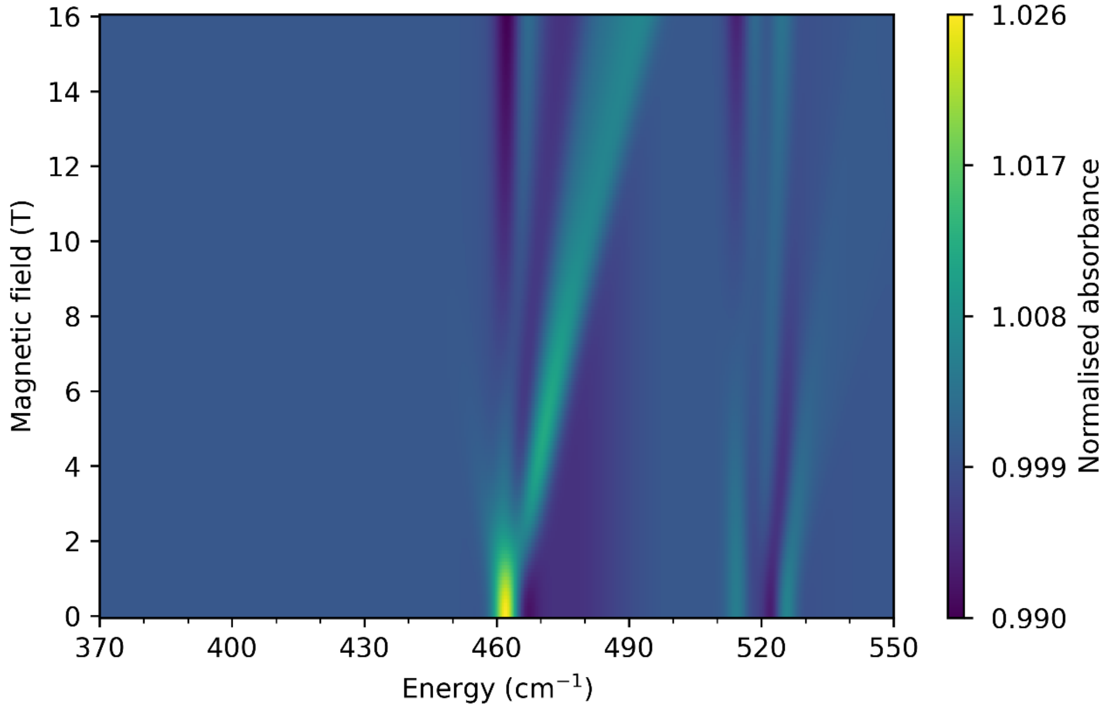

Supplementary Figure 11: Simulated FIRMS map coupling to modes 43 – 45 using CASSCF-CASPT2-SO equilibrium CFPs, CASSCF-SO spin-phonon couplings, and *ab initio* transition intensities.

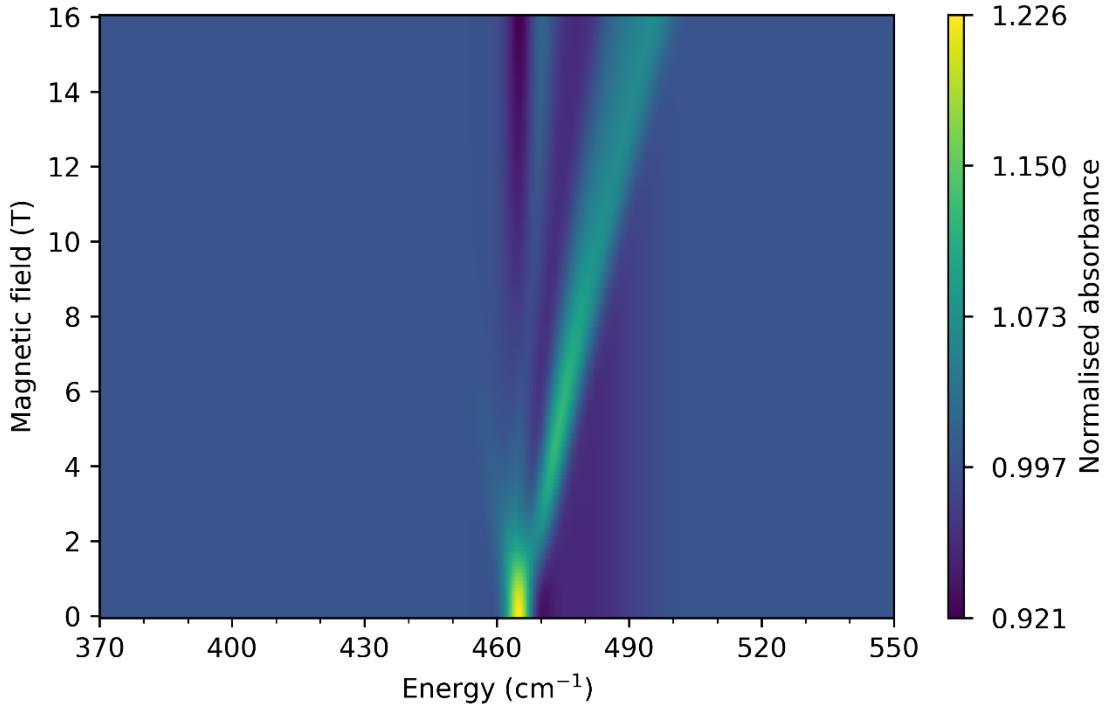

Supplementary Figure 12: Simulated FIRMS map coupling to modes 4 and 5 using CASSCF-CASPT2-SO equilibrium CFPs, CASSCF-SO spin-phonon couplings, and *ab initio* transition intensities.

Comparing the individual 3 mode simulations (Supplementary Figures 7-11) to the 9+2 mode composite simulation (Figure 3b) in the main text, we see almost identical results for the purely vibronic signals. This agreement is unsurprising, as we neglect the quadratic terms in Equation 5 which couple vibrational modes together. However, while the vibrational modes are separate from one-another, all will couple to the electronic structure simultaneously, and so we see only a single purely electronic signal ($|1_\pm\rangle \rightarrow |2_\pm\rangle, E_{B=0}$ ≈ 470-480 cm^{-1}) in our 9-mode simulation which is very close to the experimental value ($E_{B=0}$ ≈ 474 cm^{-1}) (Supplementary Figure 12).

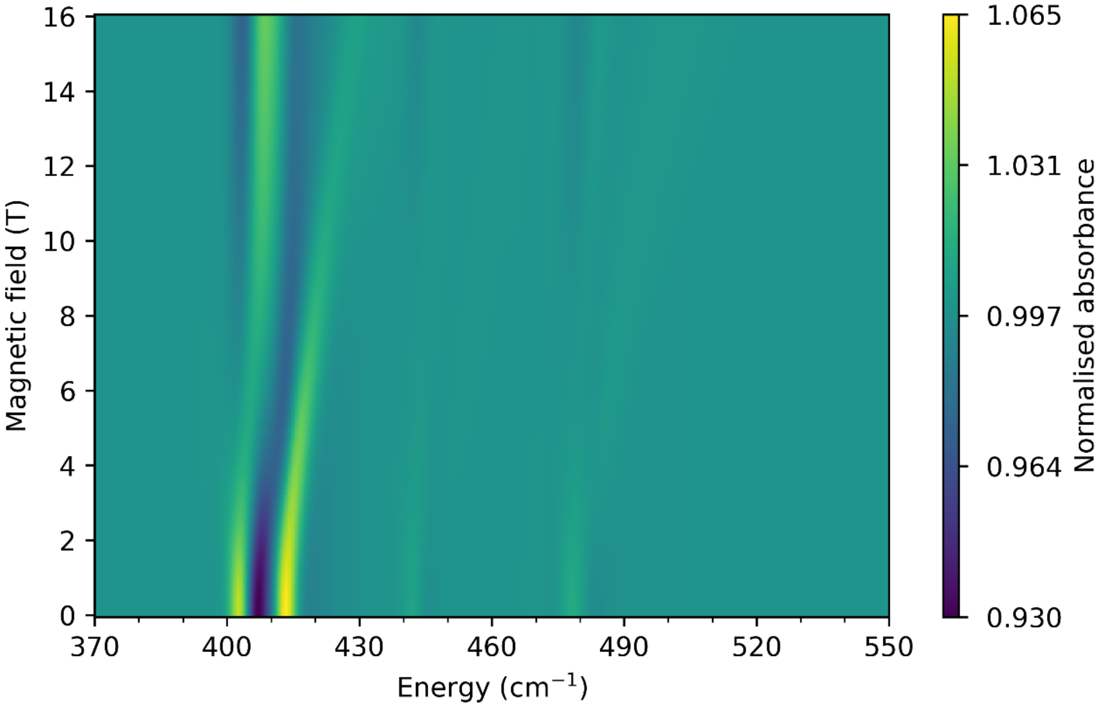

Supplementary Figure 13: Simulated FIRMS map coupling to modes 34-42 using CASSCF-CASPT2-SO equilibrium CFPs, CASSCF-SO spin-phonon couplings, and *ab initio* transition intensities.

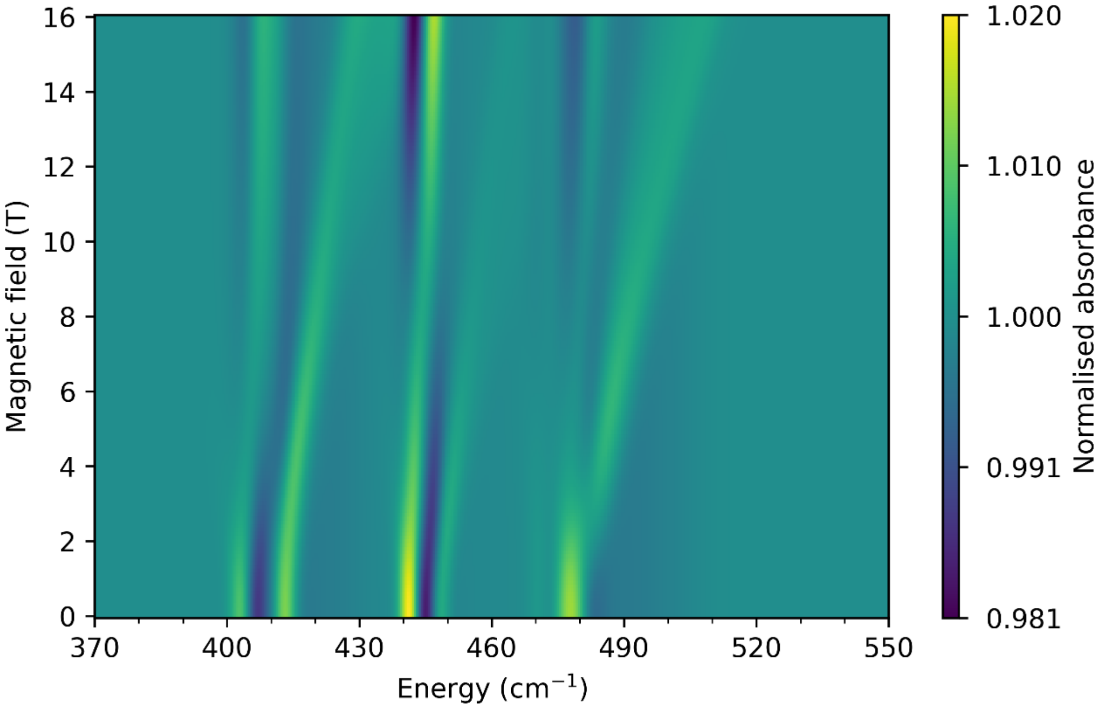

Supplementary Figure 14: Simulated FIRMS map coupling to modes 34-42 using CASSCF-CASPT2-SO equilibrium CFPs, CASSCF-SO spin-phonon couplings, and *ab initio* transition intensities in which the vibrational transition probabilities have been scaled according to Supplementary Figure 3, and the electronic transition probabilities have been scaled by 20 times.

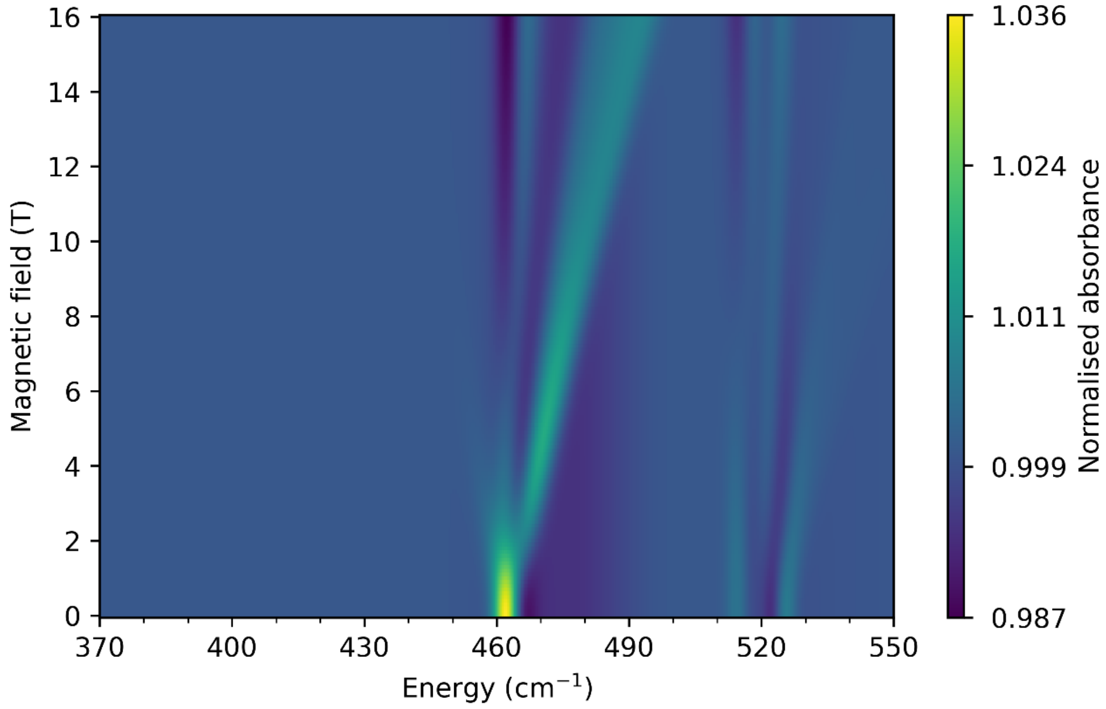

Supplementary Figure 15: Simulated FIRMS map coupling to modes 43-45 using CASSCF-CASPT2-SO equilibrium CFPs, CASSCF-SO spin-phonon couplings, and *ab initio* transition intensities in which the vibrational transition probabilities have been scaled according to Supplementary Figure 3, and the electronic transition probabilities have been scaled by 20 times.

In the higher energy range near the second excited KD, there is a very intense signal **G** ($E_{B=0}$ ≈ 775 cm^{-1}) in the experimental FIRMS map which seems almost field independent at high (> 10 T) and low (< 6 T) fields, but more field dependent at intermediate fields (Supplementary Figure 17). The shape of this signal is similar to that of the avoided crossings observed by Xue *et al.*, in which a purely electronic and field dependent transition interacts with a nearby purely vibrational transition.[5] Unfortunately, we are unable to see any signal corresponding to the nearby purely electronic $|1_{\pm}, n\rangle \rightarrow |3_{\pm}, n\rangle$ transition at zero field, as it lies in a region of low IR transmission. However, due to the relatively strong field dependence of signal **G** and its distance from the expected $|1_{\pm}, n\rangle \rightarrow |3_{\pm}, n\rangle$ transition energy (*ca.* 745 cm^{-1}), signal **G** is unlikely to be the result of an avoided crossing. Rather, we believe signal **G** is caused by an intra-Kramers vibronic transition and will have an increased signal intensity due to its proximity to the nearby electronic transition. To account for its unusual shape, we note that, much like signals **A** and **B**, signal **G** emanates from a region of high IR absorption (~770 cm^{-1}) which effectively masks any less intense field dependent signals. Indeed, tracing backwards from

the field-dependent region at 8 T (785 cm^{-1}) to 0 T gives $E_{B=0} \approx$ 765 cm^{-1}, which coincides with three modes in our DFT calculations at 762 cm^{-1} (modes 58 and 59, E, and mode 60, A, which are all out-of-plane bends of the aromatic hydrogens, Supplementary Videos 25-27), and a single mode at 768 cm^{-1} (mode 61, A, which is a Yb-NR_3 stretch, Supplementary Video 28), and thus signal **G** could result from an intra-KD transition coupled to modes 58-61. Alternatively, like signal **F**, signal **G** could be caused by an inter-KD transition $|1_{\pm},0\rangle \rightarrow |3_{\pm},1\rangle$ with a mode of *ca.* 10-30 cm^{-1}, or a $|1_{\pm},0\rangle \rightarrow |2_{\pm},1\rangle$ inter-KD transition with a mode around 290 cm^{-1}. Given the plethora of possible origins for this signal, its broad character, and its positioning adjacent to a strong IR absorption, we do not attempt to simulate this signal here.

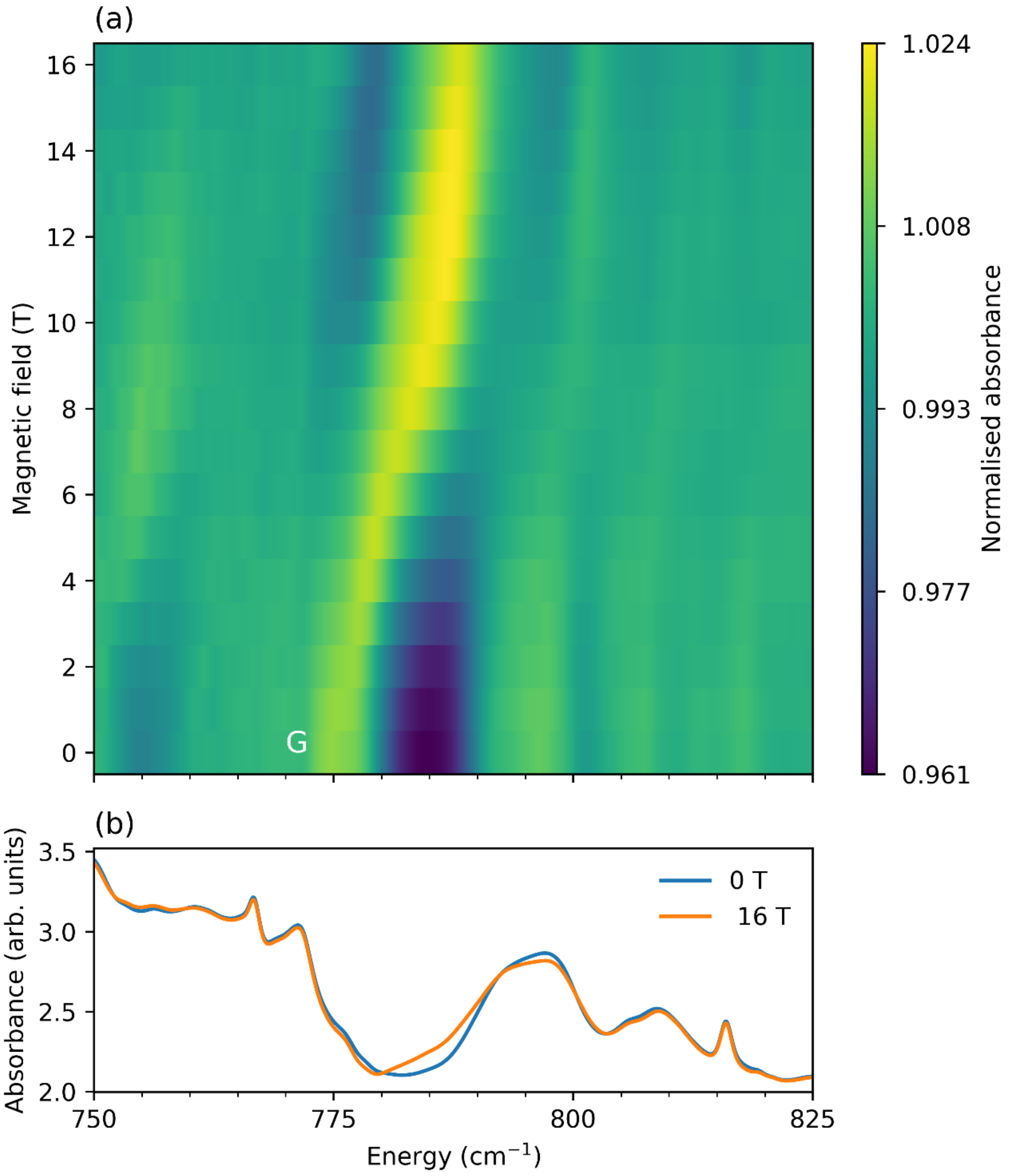

Supplementary Figure 16: (a) Experimental FIRMS map measured at 4.2 K in the range 750 to 825 cm^{-1}; field dependent signal labelled as **G**. (b) Raw FTIR spectra taken in 0 T and 16 T applied magnetic fields.

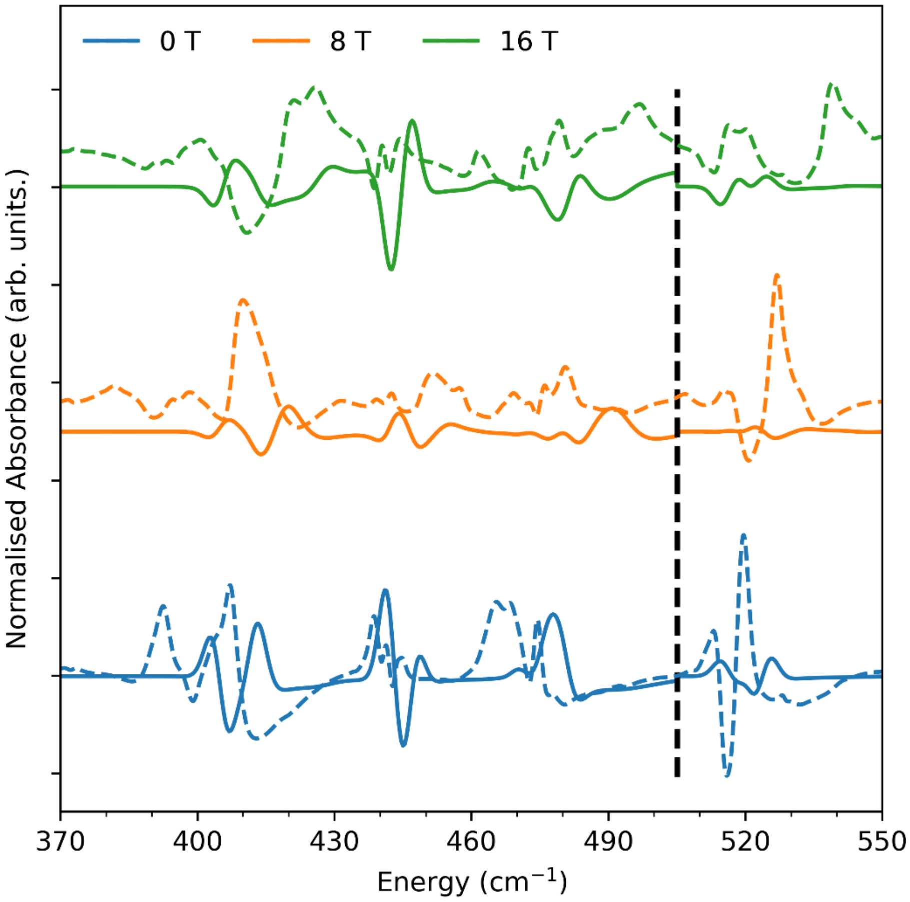

Supplementary Figure 17: Cuts of the simulated (solid lines) and experimental (dashed lines) FIRMS spectra at 0, 8 and 16 T.

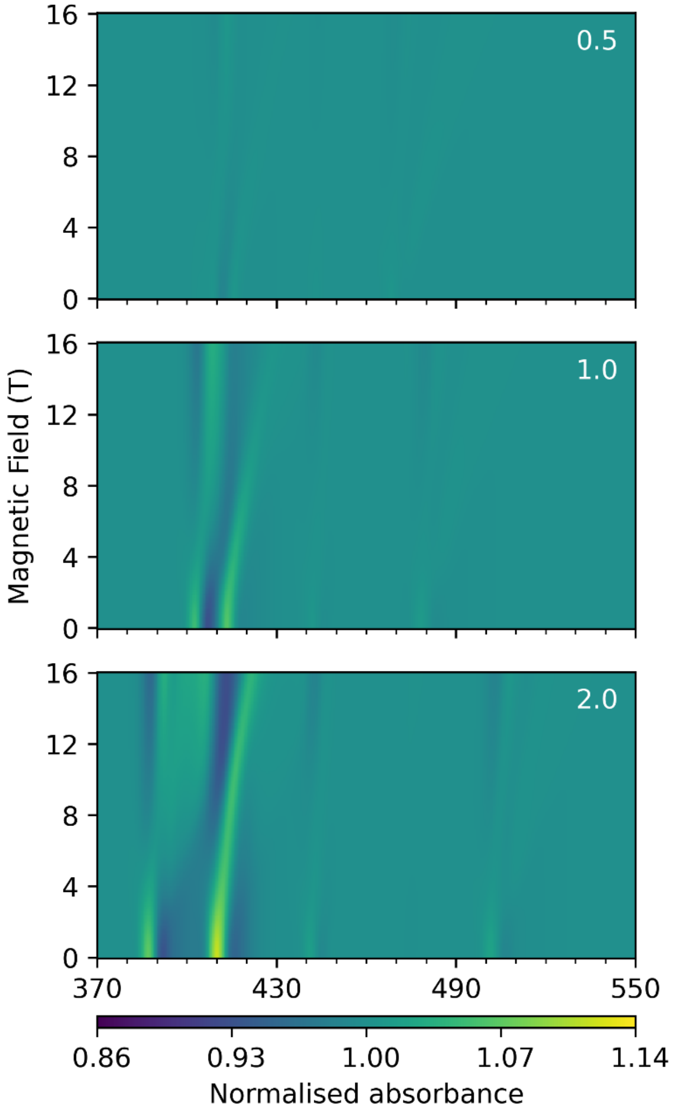

Supplementary Figure 18: Simulated FIRMS map coupling to modes 34-42, scaling each $\hat{H}_{\mathrm{coup},j}$ by either a factor of either 0.5 (top), 1.0 (middle) or 2.0 (bottom). All simulations use CASSCF-CASPT2-SO equilibrium CFPs, CASSCF-SO spin-phonon couplings, and *ab initio* transition intensities with no scaling of the vibrational or electronic transition probabilities.

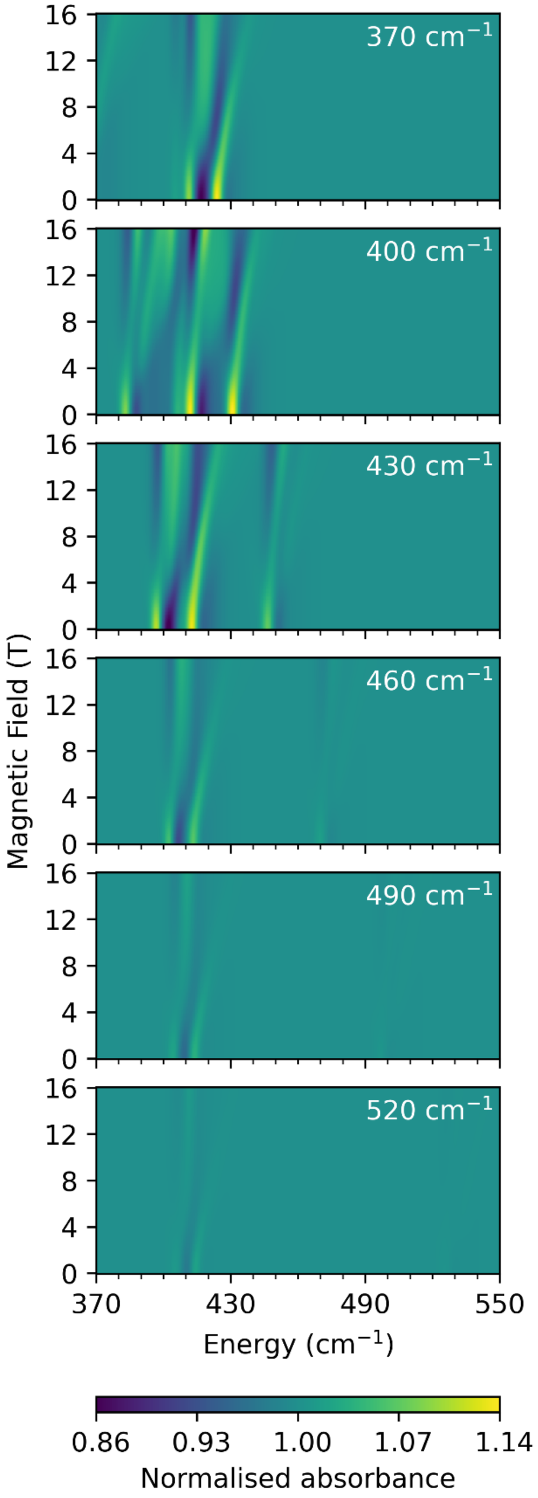

Supplementary Figure 19: Simulated FIRMS map coupling to modes 34-36, resetting the $|1_{\pm},0\rangle \rightarrow |2_{\pm},0\rangle$ electronic energy to: 370, 400, 430, 460, 490, and 520 cm^{-1} (white text). Simulations use CASSCF-CASPT2-SO equilibrium CFPs, CASSCF-SO spin-phonon couplings, and *ab initio* transition intensities in which the vibrational transition probabilities have been scaled according to Supplementary Figure 3, and the electronic transition probabilities have been scaled by 20 times.

In the lower energy range, there are weak signals in the experimental FIRMS map at 167 cm^{-1}, 238 cm^{-1}, 553 cm^{-1}, 581 cm^{-1}, all of which are distant from any electronic transitions (Supplementary Figures 19 and 20). Of these, the former two are clearly present in the luminescence measurements (Peaks 1a and 1c, Figure 2) and are intra-KD vibronic transitions at energies which match those of vibrational modes from our DFT calculations (Supplementary Table 6). The energies of the latter two signals match well with vibrational modes from DFT and could be intra-KD signals. However, as they are also above the energy of the first excited electronic state they could also be inter-KD transitions involving low energy modes which are also present in our DFT results.

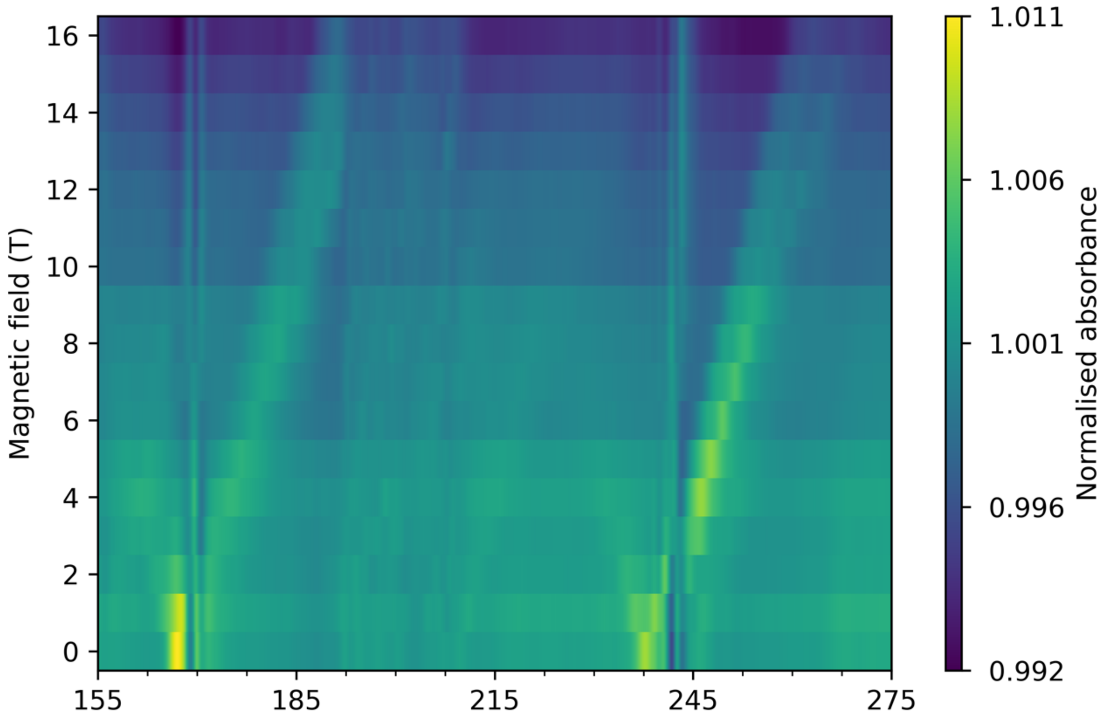

Supplementary Figure 20: Experimental FIRMS map measured at 4.2 K in the range 155-275 cm^{-1}.

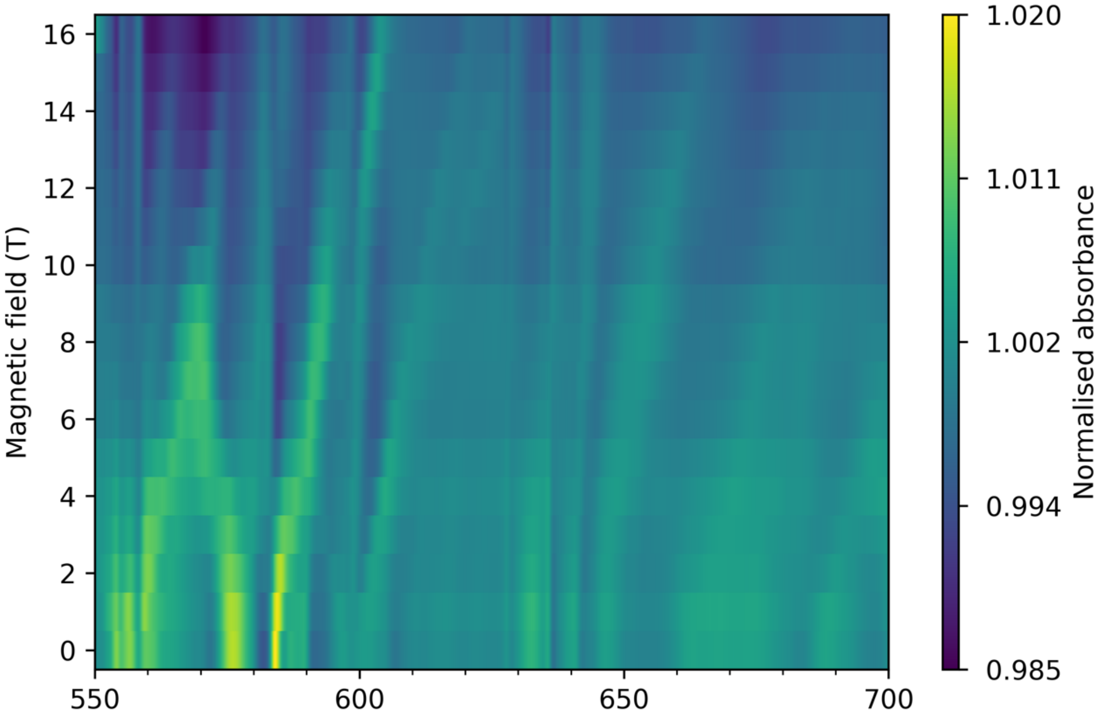

Supplementary Figure 21: Experimental FIRMS map measured at 4.2 K in the range 550-700 cm^{-1}.

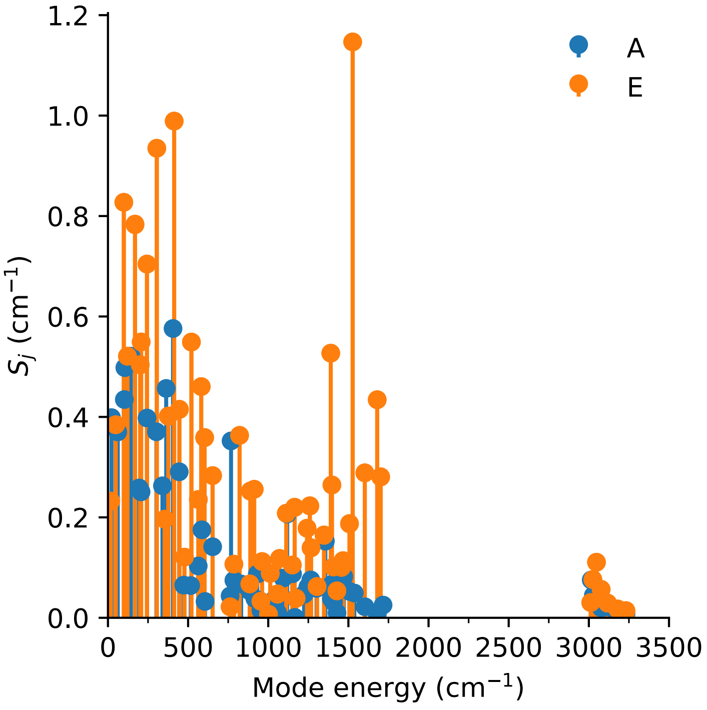

Supplementary Figure 22: *Ab initio* calculated vibronic coupling strength S_j of modes of $\mathbf{1}_{\mathbf{opt}}$ with A (blue) and E (orange) symmetry.

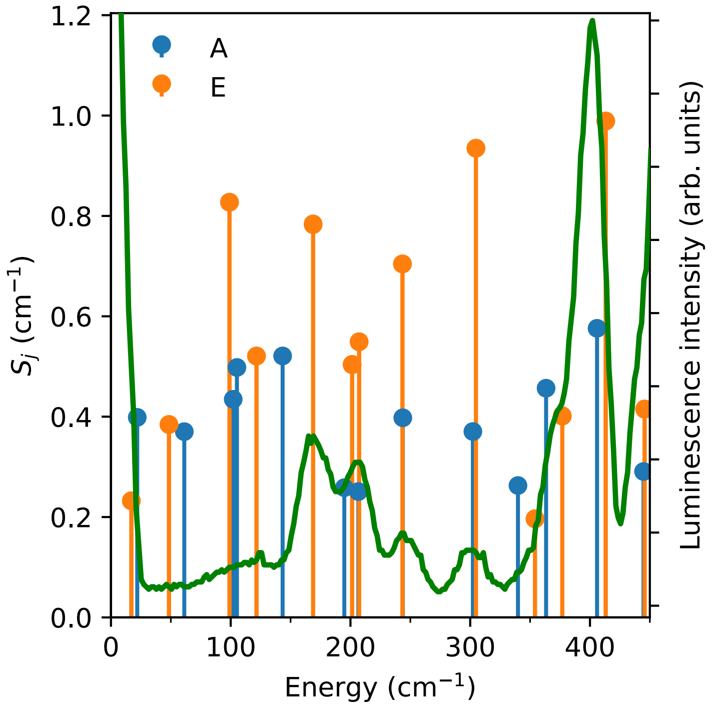

Supplementary Figure 23: Ab initio calculated vibronic coupling strength S_j of modes of $\mathbf{1}_{\mathbf{opt}}$ with A (blue) and E (orange) symmetry superimposed onto the luminescence spectrum (green) of **1** in the region 0 cm^{-1} to 450 cm^{-1}.

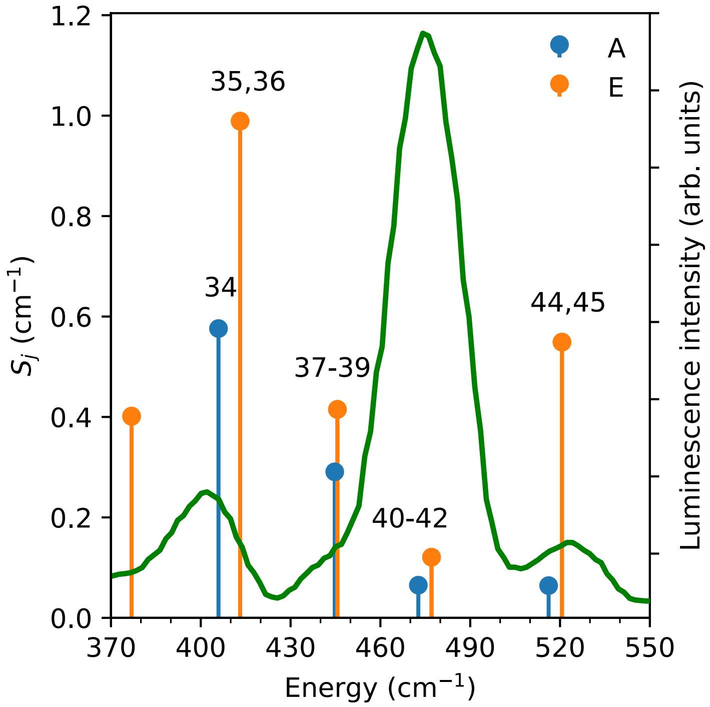

Supplementary Figure 24: Ab initio calculated vibronic coupling strength S_j of modes of $\mathbf{1}_{\mathbf{opt}}$ with A (blue) and E (orange) symmetry superimposed onto the luminescence spectrum (green) of **1** in the region 370 cm^{-1} to 550 cm^{-1}.

References

1 K. S. Pedersen, J. Dreiser, H. Weihe, R. Sibille, H. V. Johannesen, M. A. Sørensen, B. E. Nielsen, M. Sigrist, H. Mutka, S. Rols, J. Bendix and S. Piligkos, *Inorg. Chem.*, 2015, **54**, 7600–7606.

2 J. Neugebauer, M. Reiher, C. Kind and B. A. Hess, *J. Comput. Chem.*, 2002, **23**, 895–910.

3 M. Eden and M. H. Levitt, *J. Magn. Res.*, 1998, **132**, 220–239.

4 J. Nehrkorn, A. Schnegg, K. Holldack and S. Stoll, *Phys. Rev. Lett.*, 2015, **114**, 010801.

5 D. H. Moseley, S. E. Stavretis, K. Thirunavukkuarasu, M. Ozerov, Y. Cheng, L. L. Daemen, J. Ludwig, Z. Lu, D. Smirnov, C. M. Brown, A. Pandey, A. J. Ramirez-Cuesta, A. C. Lamb, M. Atanasov, E. Bill, F. Neese and Z. L. Xue, *Nat. Commun.*, 2018, **9**, 2572.
