# Supplementary material for: Analysis of vibronic coupling in a 4f molecular magnet with FIRMS
Source: Nat Commun. 2022 Feb 11;13:825. doi: 10.1038/s41467-022-28352-2 (PMC8837795; doi:10.1038/s41467-022-28352-2)
Supplement: Supplementary file 32 — Description of Additional Supplementary Files [file 41467_2022_28352_MOESM32_ESM.pdf]

Title: Supplementary Movie 1
Description: Vibrational mode 4

Title: Supplementary Movie 2
Description: Vibrational mode 5

Title: Supplementary Movie 3
Description: Vibrational mode 7

Title: Supplementary Movie 4
Description: Vibrational mode 8

Title: Supplementary Movie 5
Description: Vibrational mode 14

Title: Supplementary Movie 6
Description: Vibrational mode 15

Title: Supplementary Movie 7
Description: Vibrational mode 20

Title: Supplementary Movie 8
Description: Vibrational mode 21

Title: Supplementary Movie 9
Description: Vibrational mode 22

Title: Supplementary Movie 10
Description: Vibrational mode 23

Title: Supplementary Movie 11
Description: Vibrational mode 26

Title: Supplementary Movie 12
Description: Vibrational mode 27

Title: Supplementary Movie 13
Description: Vibrational mode 34

Title: Supplementary Movie 14
Description: Vibrational mode 35

Title: Supplementary Movie 15
Description: Vibrational mode 36

Title: Supplementary Movie 16
Description: Vibrational mode 37

Title: Supplementary Movie 17
Description: Vibrational mode 38

Title: Supplementary Movie 18
Description: Vibrational mode 39

Title: Supplementary Movie 19
Description: Vibrational mode 40

Title: Supplementary Movie 20
Description: Vibrational mode 41

Title: Supplementary Movie 21
Description: Vibrational mode 42

Title: Supplementary Movie 22
Description: Vibrational mode 43

Title: Supplementary Movie 23
Description: Vibrational mode 44

Title: Supplementary Movie 24
Description: Vibrational mode 45

Title: Supplementary Movie 25
Description: Vibrational mode 58

Title: Supplementary Movie 26
Description: Vibrational mode 59

Title: Supplementary Movie 27
Description: Vibrational mode 60

Title: Supplementary Movie 28
Description: Vibrational mode 61

Title: Supplementary Movie 29
Description: Vibrational mode 142

Title: Supplementary Movie 30
Description: Vibrational mode 143
